# Preferential binding of HIF-1 to transcriptionally active loci determines cell-type specific response to hypoxia

**DOI:** 10.1186/gb-2009-10-10-r113

**Published:** 2009-10-14

**Authors:** Xiaobo Xia, Andrew L Kung

**Affiliations:** 1 Department of Pediatric Oncology, Dana-Farber Cancer Institute, Children's Hospital Boston, and Harvard Medical School, Binney Street, Boston, MA 02115, USA

## Abstract

**Background:**

Hypoxia-inducible factor 1 (HIF-1) plays a key role in cellular adaptation to hypoxia. To better understand the determinants of HIF-1 binding and transactivation, we used ChIP-chip and gene expression profiling to define the relationship between the epigenetic landscape, sites of HIF-1 binding, and genes transactivated by hypoxia in two cell lines.

**Results:**

We found that when cells were acutely subjected to hypoxia, HIF-1 preferentially bound to loci that were already transcriptionally active under normal growth conditions characterized by the presence of histone H3 lysine 4 methylation, the presence of RNA polymerase II, and basal production of mRNA. Cell type-specific differences in HIF-1 binding were largely attributable to differences in the basal gene expression patterns in the cells prior to the onset of hypoxia.

**Conclusions:**

These results suggest that the repertoire of genes active in a cell (for example, through lineage specific transcription factors) defines the subset of genes that are permissive for binding and transactivation by stimulus-responsive transcription factors.

## Abstract 2

ChIP-chip and microarray expression studies show that, in response to hypoxia, HIF-1 preferentially binds to and up-regulates already active genes.

## Background

Hypoxia, a reduction in the normal level of oxygen in tissues, occurs during various physiological and pathological conditions, such as embryonic development, ischemic disease, pulmonary disease, and cancer [1]. The transcription factor Hypoxia-inducible factor 1 (HIF-1) is a key mediator of cellular homeostasis in response to hypoxia. HIF-1 transactivates genes that facilitate metabolic adaptation by shifting from oxidative phosphorylation to anaerobic glycolysis, and enhances oxygen delivery by inducing vasodilatation, increasing vascular permeability, enhancing erythropoiesis, and angiogenesis [1]. Our previous studies have also suggested a third compensatory program consisting of up-regulation of multiple members of the 2-OG-dioxygenase family, which all require molecular oxygen for their enzymatic activity [2].

Several hundreds of genes have been validated as direct targets of HIF-1 transactivation in a variety of biological systems [2-4]. Alignment of the sequences encompassing these HIF-1 binding sites has revealed a consensus core motif of 5'-A/GCGTG-3'. However, it is clear that this promiscuous motif cannot be the sole determinant of HIF-1 binding and transactivation. As is the case for other transcription factors such as E2F1, Myc, estrogen receptor, FoxA1, and p63 [5-8], HIF-1 binds to only a small proportion of predicted binding sites under hypoxic conditions [2,4], although the basis for selectivity is incompletely understood.

The binding of certain transcription factors to chromatin can be modulated by DNA methylation - for example, Myc and CREB binding is precluded by methylation of their cognate DNA binding sites [9,10]. Previous studies have demonstrated that HIF-1 binding to the 3' enhancer of the erythropoietin (*EPO*) gene is also modulated by methylation of the hypoxia response element within the enhancer [11,12]. Expression of *EPO *is restricted to cell types in which the hypoxia response element is unmethylated. Furthermore, expression of the HIF-1 target BNIP3 is selectively silenced by histone deacetylation and methylation in colorectal cancer [13]. Together, these single-locus studies suggest that epigenetic modifications may, in part, modulate the binding of HIF-1 to chromatin and subsequent gene transactivation.

Gene expression profiling studies have revealed thousands of genes whose expression changes with hypoxia, with vast differences between cell types in the specific genes induced [14-21]. In previous studies we used chromatin immunoprecipitation (ChIP) coupled with analysis on tiled microarrays (ChIP-chip) to identify HIF-1 binding sites across the human genome in HepG2 cells [2]. When coupled with gene expression profiling, our studies revealed hundreds of primary targets of HIF-1 transactivation in this cell type. To more broadly understand the basis for the selectivity of HIF-1 binding and cell-type-specific differences in response to hypoxia, in the current study we assessed HIF-1 binding in a second cell type, U87 glioma cells, and assessed the epigenetic landscape across the genome of these two cell types. We integrated these results with gene expression profiles to elucidate the determinants of HIF-1 binding, transactivation, and cell type specificity.

## Results

### HIF-1 binds to transcriptionally active genes

**Figure 1:**
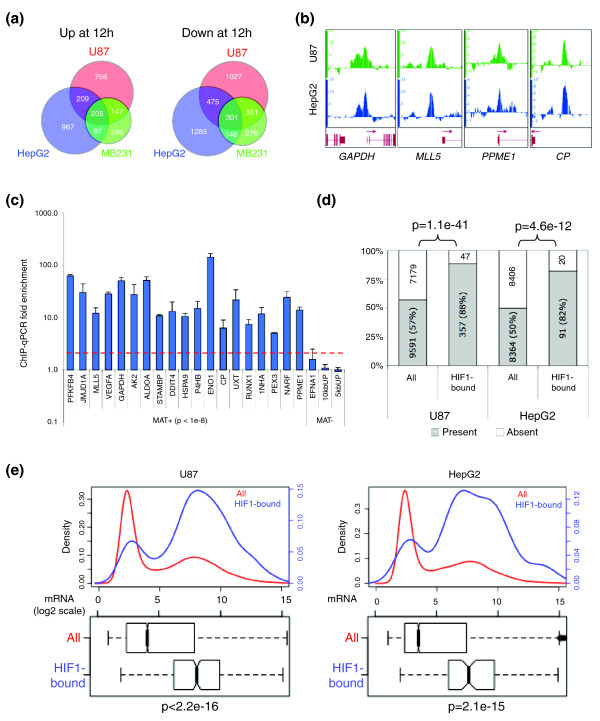
HIF-1 preferentially binds to promoters of transcriptionally active genes. **(a) **Proportional Venn diagrams of genes with mRNA expression significantly (*P*-value < 0.01) up- or down-regulated after 12 h of hypoxia in U87, HepG2, and MDA-MB231 cells. **(b) **Results of HIF-1 ChIP-chip analysis on promoter arrays (U87) was highly similar to analysis on whole genome arrays (HepG2). Representative Integrated Genome Browser tracks are shown with the same scale for both cell types. **(c) **ChIP-quantitative PCR validation of U87 HIF-1 ChIP hits. Data expressed as fold enrichment relative to input (mean ± standard deviation of independent replicates). An increase of more than two-fold (red dashed line) was considered positive for HIF-1 binding. 10 kbUP and 5 kbUP are negative control regions. **(d) **Approximately half of all genes in the genome are expressed (Present MAS5 call) and half are not expressed (Absent MAS5 call) under normal growth conditions (All) in both cell lines. Upon stabilization by hypoxia, HIF-1 preferentially binds (HIF1-bound) to the promoter of genes that are present under normal growth conditions. Statistical significance determined by Fisher exact test. **(e) **Genes bound by HIF-1 in U87 or HepG2 cells (HIF1-bound) have higher levels of basal mRNA expression than the normal distribution of all genes (All). Top panel: density plots of genes at indicated mRNA levels. Bottom panel: box plot of all genes (All) compared to genes bound by HIF-1 (HIF1-bound). Statistical significance determined by Student's *t*-test. For box plots, the median is indicated by a dark bar, the box bounds the lower and upper quartiles, the whiskers define the data range, and the notches represent the 95% confidence interval.

The subsets of genes induced by hypoxia vary greatly amongst different cell types. Some of these differences may be due to variations in culture conditions, length of exposure to hypoxia, degree of hypoxia, and microarray platforms. However, even after standardizing all of these variables, we verified by gene expression profiling that most hypoxia-induced changes in mRNA expression were cell type specific (Figure 1a). When comparing the genes induced or repressed by hypoxia in HepG2 hepatoma cells, U87 glioma cells and MDA-MB231 breast cancer cells, only a minority of all genes were concordantly up- or down-regulated across all three cell types (Figure 1a).

To better understand HIF-1 binding and transactivation, we previously identified HIF-1 binding sites across the human genome in HepG2 cells by ChIP-chip [2]. To determine if some of the cell-type specific responses in gene expression (Figure 1a) under hypoxia resulted from differential HIF-1 binding, we used ChIP-chip to identify HIF-1 binding sites in U87 glioma cells. Since a majority of HIF-1 binding sites in HepG2 cells were within promoter regions [2], we analyzed U87 HIF-1 ChIP samples on tiled arrays covering approximately 10 kb surrounding the transcriptional start sites (TSS) of all known genes. We used the Model-based Analysis of Tiling-array (MAT) algorithm [22] to identify HIF-1 binding sites comparing triplicate hypoxic (0.5% O_2_, 4 h) to triplicate normoxic samples. Peaks of probe intensity were morphologically similar comparing previous whole genome (HepG2) data to the current promoter array (U87) data (Figure 1b). To ensure specificity, we used a stringent cutoff (*P*-value < 1 × 10^-8^) above which all loci checked by quantitative PCR (qPCR) were true positives (Figure 1c). With this cutoff, 387 binding loci were identified as HIF-1 binding sites in U87 cells (Additional data file 1). We used gene set enrichment analysis (GSEA) [23] to determine whether HIF-1 binding was associated with altered gene expression under conditions of hypoxia. Similar to what we found for HepG2 cells [2], HIF-1 bound genes were highly associated with up-regulated gene expression under hypoxic conditions (nominal *P*-value and false discovery rate q-value < 0.001; Additional data file 2).

To enable comparison between the two cell types and to ensure specificity, the same stringent cutoff was applied to HIF-1 binding sites previously identified in HepG2 cells [2]. Furthermore, HIF-1 binding sites in the HepG2 dataset were restricted to those that mapped to probes represented on the promoter arrays used in this study. Among 201 HepG2 HIF-1 binding sites that were above this cutoff, 117 were in regions represented on the promoter arrays.

When we integrated sites of HIF-1 binding (after 4 h of hypoxia) with gene expression profiles over a time course of hypoxia (0, 4, 8 and 12 h of hypoxia), we noted that loci that were bound by HIF-1 were biased towards genes that were already active prior to induction of hypoxia. Under normal growth conditions (t = 0 h), there were roughly equal numbers of genes with and without basal mRNA production ('present' and 'absent' MAS5 calls) in each cell type (Figure 1d, 'All'). However, most genes bound by HIF-1 (82% and 88%) in each cell type had present calls prior to the onset of hypoxia (Figure 1d, 'HIF1-bound'). Consistent with this, the basal expression levels of all genes had a bimodal distribution in both cell types (Figure 1e, 'All'), but the distribution of genes bound by HIF-1 was significantly skewed towards higher levels of basal expression (Figure 1e, 'HIF1-bound'). Together, these results demonstrate that when HIF-1 is acutely stabilized by hypoxia (4 h), there is a striking bias for its binding to loci that were already transcriptionally active under normal growth conditions (prior to onset of hypoxia).

### HIF-1 preferentially binds to transcriptionally active loci

**Figure 2:**
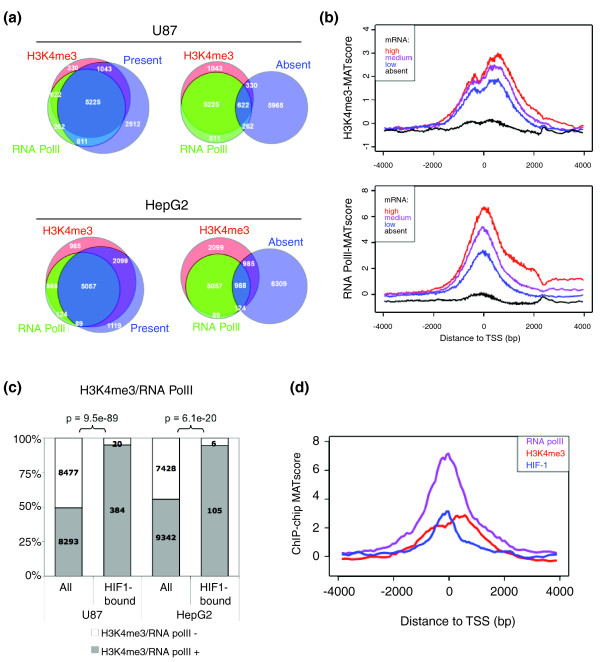
Determinants of HIF-1 binding. **(a) **H3K4 me3 modification and RNA Pol II binding at promoters are highly correlated with basal mRNA expression (present call). Proportional Venn diagrams of H3K4 me3 mark, RNA Pol II binding, and present calls in normal growth conditions in U87 and HepG2 cells. **(b) **mRNA expression levels are positively correlated with both H3K4 me3 and RNA Pol II binding intensities. All genes were separated into groups based on mRNA expression levels. Probe level intensities for H3K4 me3 and RNA Pol II were plotted as the aggregated mean of all genes in each group relative to the TSS. **(c) **Approximately half of all genes are marked with H3K4 me3 or the presence of RNA Pol II under normal growth conditions (All). Nearly all loci bound by HIF-1 (HIF1-bound) are marked by H3K4 me3 or RNA Pol II. Statistical significance determined by Fisher exact test. **(d) **Aggregate probe level intensities for H3K4 me3 (red), RNA Pol II (purple), and HIF-1 (blue) for all HIF-1 bound promoters are plotted relative to the TSS.

Since histone H3 trimethyl-lysine 4 (H3K4 me3) modification and the presence of RNA polymerase II (RNA Pol II) are associated with active promoters [24,25], we used ChIP-chip to assess H3K4 me3 modifications and RNA Pol II occupancy in promoter regions in both normoxic U87 and HepG2 cells and compared their distribution with that of the HIF-1 binding sites. We identified 7,536 non-repeat binding regions for H3K4 me3 and 7,513 for RNA Pol II in U87 cells. For HepG2 cells, 10,082 non-repeat binding regions were identified for H3K4 me3 and 7,333 for RNA Pol II. Consistent with previous findings [26], in both cell types genes with mRNA production (present MAS5 call) were strongly associated with the presence of H3K4 me3 and RNA Pol II, whereas genes without mRNA production (absent MAS5 call) had a counter-relationship with these marks (Figure 2a). On a gene-specific level, the amount of H3K4 me3 modification and RNA Pol II binding were strongly correlated with the level of mRNA expression from the locus (Figure 2b; Additional data file 3).

The promoters bound by HIF-1 (after 4 h of hypoxia) were characterized by H3K4 me3 and RNA Pol II occupancy under normal growth conditions prior to the onset of hypoxia (Additional data file 3). In both U87 and HepG2 cells, almost all promoters bound by HIF-1 (95.0% for U87 and 94.6% for HepG2) were positive for either H3K4 me3 or RNA Pol II under basal conditions (Figure 2c, 'HIF1-bound'), which is significantly skewed in comparison to the normal distribution of all genes (Figure 2c, 'All'). The distribution of HIF-1 and RNA Pol II binding sites were nearly identical, centered just before the TSS (Figure 2d). In contrast, the distribution of H3K4 me3 had a small dip at the TSS, consistent with prior observations that activated promoters are characterized by nucleasome-poor regions around the TSS [27,28].

In the minority of cases where HIF-1 bound to a gene with an absent call, we usually found H3K4 me3 and/or RNA Pol II present in the promoter despite the absent call (38 out 47 for U87, 18 out 20 for HepG2). This is consistent with previous reports that, in both embryonic stem cells and differentiated cells, many genes show signs of transcriptional initiation (for example, positive RNA Pol II) but produce no full length transcripts (for example, absent call) [29]. These genes are thought to be poised for activation and inducible genes that can respond rapidly upon particular stimulation. Only a small minority (approximately 2%) of the HIF-1 bound genes (9 out of 404 for U87 and 2 out of 111 for HepG2) had no evidence of activation (no H3K4 me3 modification, no RNA Pol II occupancy, and an Absent call).

Together, these data indicate that, in both cell types, HIF-1 preferentially binds to loci that were already transcriptionally active under normal growth conditions as indicated by the presence of RNA Pol II, H3K4 me3 modification, and basal mRNA production.

### Cell-type specific differences in HIF-1 binding

Since HIF-1 preferentially binds to transcriptionally active loci, we wondered whether cell-type-specific differences in gene expression might underlie differences in HIF-1 binding. We first compared HIF-1 binding between U87 and HepG2 cells. For HepG2 HIF-1 sites that were represented on promoter arrays, more than half (72 out of 117) were bound by HIF-1 at the identical site in both cell lines under stringent conditions (Additional data file 4). Only 24 sites bound by HIF-1 in HepG2 cells had no evidence of HIF-1 binding in U87 cells at any stringency, and these were considered HepG2-unique binding sites.

**Figure 3:**
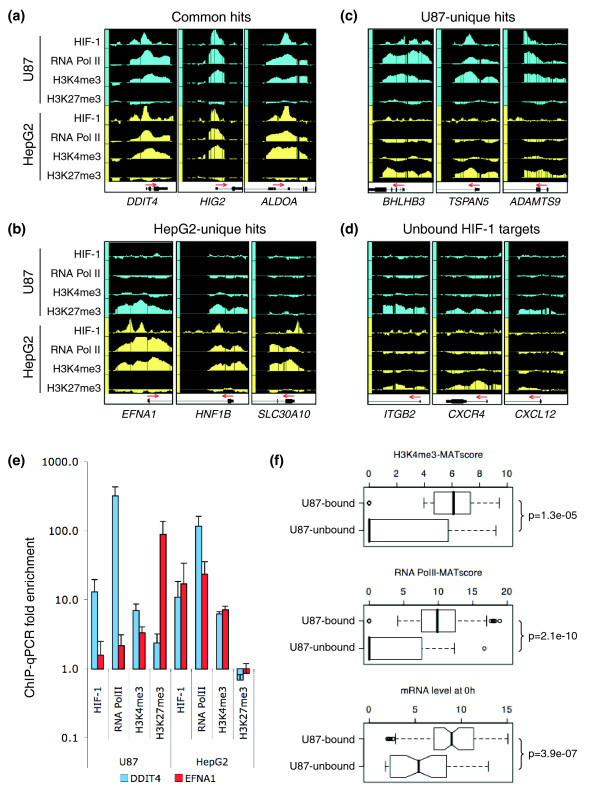
Cell-type specific gene expression predicts HIF-1 binding. For the indicated genes, IGB tracks for HIF-1, RNA Pol II, H3K4 me3 and H3K27 me3 are shown with identical scales between cell types. Representative data are shown for **(a) **HIF-1 hits common to both cell types, **(b) **HIF-1 ChIP hits unique to HepG2 or **(c) **U87 cells, and **(d) **HIF-1 binding sites reported in the literature but not bound in either cell type. **(e) **ChIP-qPCR analysis of HIF-1, H3K4 me3, RNA Pol II, and H3K27 me3 at the indicated loci. For HIF-1, H3K4 me3, and RNA Pol II ChIP, results are normalized to negative control regions located 5 kb and 10 kb upstream of the vascular endothelial growth factor (*VEGF*) gene. For H3K27 me3 ChIP, results are normalized to the promoter regions of the glyceraldehyde 3-phosphate dehydrogenase (*GAPDH*) and aldolase A (*ALDOA*) genes. Data expressed as mean ± SD of independent replicates. **(f) **A set of well-validated HIF-1 target genes were partitioned based on whether HIF-1 binding was observed (U87-bound) or absent (U87-unbound) in U87 cells. Binding of HIF-1 is highly correlated with H3K4 me3 modification, RNA Pol II occupancy, and basal mRNA production. Statistical significance was determined by Student's *t*-test. For box plots, the median is indicated by a dark bar, the box bounds the lower and upper quartiles, the whiskers define the data range, and the notches represent the 95% confidence interval.

The sites that were similarly bound by HIF-1 in both cell lines were characterized by H3K4 me3 and RNA Pol II occupancy in both cell lines (for example, *DDIT4*; Figure 3a). In the case of loci in which HIF-1 binding was discordant between the two cell lines, H3K4 me3 and RNA Pol II occupancy usually predict the binding of HIF-1. Sites that were bound by HIF-1 only in HepG2 cells were characterized by the presence of RNA Pol II and H3K4 me3 modification in HepG2 but not U87 cells (for example, *EFNA1*; Figure 3b). The converse pattern was also observed for HIF-1 binding sites specific to U87 cells (for example, *BHLHB3*; Figure 3c). In addition, among previously well-characterized HIF-1 bound loci [3] in which we did not observe HIF-1 binding in either cell line, H3K4 me3 and RNA Pol II were generally absent in the basal state (Figure 3d). Although we also performed ChIP-chip analysis of the repressive histone H3 trimethyl-lysine 27 (H3K27 me3) modification, the signal enrichment above input on the arrays was too weak for us to feel confident that we had captured a sensitive representation of this epigenetic mark. Nevertheless, at loci where the H3K27 me3 signal was positive, there was usually an inverse relationship with RNA Pol II occupancy, H3K4 me3 modification and HIF-1 binding (for example, *EFNA1 *and *BHLHB3*; Figure 3b, c). These results were verified at representative loci using ChIP-qPCR (Figure 3e), and in all cases ChIP-qPCR results were concordant with the ChIP-chip results.

To further analyze cell-type-specific binding, we next examined a set of 124 previously well-validated HIF-1 bound sites composed of both high confidence binding sites found in HepG2 cells [2] and well validated HIF-1 targets identified in other cell types [3]. For this set of known HIF-1 binding sites, 77 loci were bound by HIF-1 in U87 cells, whereas 47 loci did not have HIF-1 binding. Loci in which HIF-1 binding was observed were characterized by high H3K4 me3, the presence of RNA Pol II, and higher basal mRNA production by comparison to loci in which HIF-1 binding was not observed (Figure 3f). Together, these data demonstrate that although HIF-1 is similarly stabilized in these two cell lines, the patterns of binding only partially overlap, and that cell-type-specific differences in the epigenetic landscape and basal gene expression underlie cell-type-specific differences in HIF-1 binding.

### Basal expression status determines response to hypoxia

**Figure 4:**
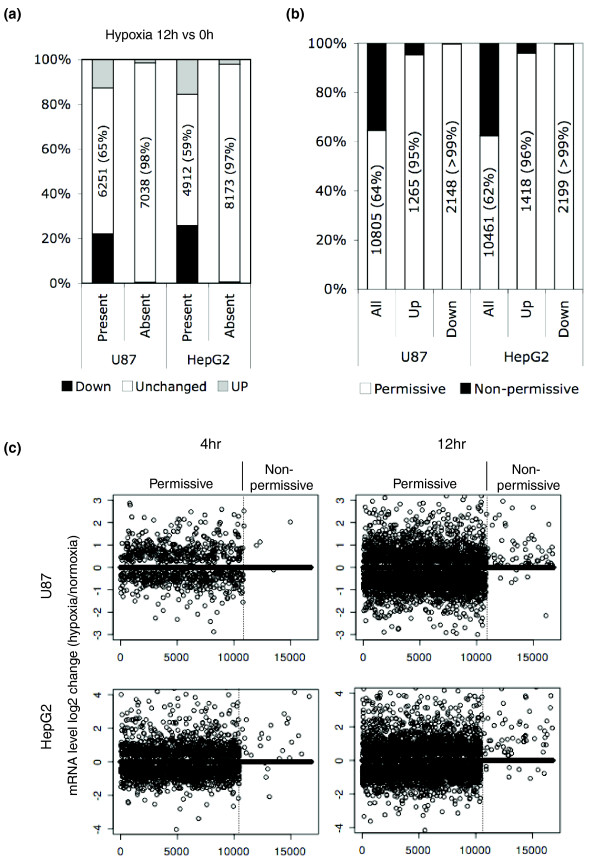
Basal expression level predicts hypoxia-inducibility. **(a) **Genes were divided based on their MAS5 present/absent calls under normoxic conditions (0 h). In both cell types, most genes whose expression was up- (gray) or down-regulated (black) by hypoxia were already expressed under basal conditions (Present). **(b) **Approximately 60% of all genes are permissive (H3K4 me3+, RNA Pol II+, or MAS5 present) under normal growth conditions (All, normoxia). Most genes for which mRNA levels were significantly up- or down-regulated upon hypoxia were permissive under normoxia. Statistical significance was determined by Fisher exact test, and was *P*-value <1e-150 for all pairwise comparisons. **(c) **For both cell types, genes were partitioned as either permissive or non-permissive under normal growth conditions (t = 0 h). Changes in mRNA levels (log2 scale) after 4 h and 12 h of hypoxia treatment are plotted, with non-significant changes (*P*-value > 0.01) represented as 0.

Although thousands of genes have altered expression under hypoxia (Figure 1a), we have only identified a few hundred direct HIF-1 targets. Therefore, a large proportion of hypoxia-induced transcriptional changes are mediated through secondary mechanisms (for example, transcription factors activated by HIF-1) or HIF-1-independent pathways. We hypothesized that the finding that HIF-1 binds to transcriptionally active loci upon activation may be generalized to many or most other transcription factors. As such, we predicted that genes that have altered expression under hypoxia (inclusive of primary HIF targets, secondary targets, and HIF-independent genes) would be those that were already transcriptionally active under normal growth conditions. Indeed, when genes were partitioned as absent or present by MAS5 call under basal growth conditions, it was clear that the absent genes very rarely changed upon hypoxia compared to the present genes (Figure 4a). For example, in U87 cells the expression of 35% of all genes that were present under normoxic conditions (t = 0 h) were either up- or down- regulated after 12 h of hypoxia treatment. However, <2% of all absent genes had expression changes upon induction of hypoxia (Figure 4a).

As noted above, in some cases genes can be in a transcriptionally permissive state with H3K4 me3 modification and/or RNA Pol II occupancy on the promoter, but without being actively transcribed (absent MAS5 call). To further investigate the underlying mechanism for selective gene response upon hypoxia, we partitioned all genes into 'permissive' or 'non-permissive' groups. The permissive group contained genes with H3K4 me3 modification, RNA Pol II occupancy, or transcribed mRNA (present MAS5 call). In contrast, the 'non-permissive' group contained genes that were negative for H3K4 me3, RNA Pol II, and mRNA production. Upon hypoxia, >95% of all up-regulated genes and >99% of all down-regulated genes in both U87 and HepG2 cells were permissive before the onset of hypoxia (Figure 4b). The rapidity and magnitude of changes in expression were also far more dramatic in permissive genes compared to non-permissive genes (Figure 4c). These results support the notion that, upon hypoxia, HIF-1 and other transcription factors are biased towards binding to and transactivating (and transrepressing) loci that are already active under normal growth conditions.

**Figure 5:**
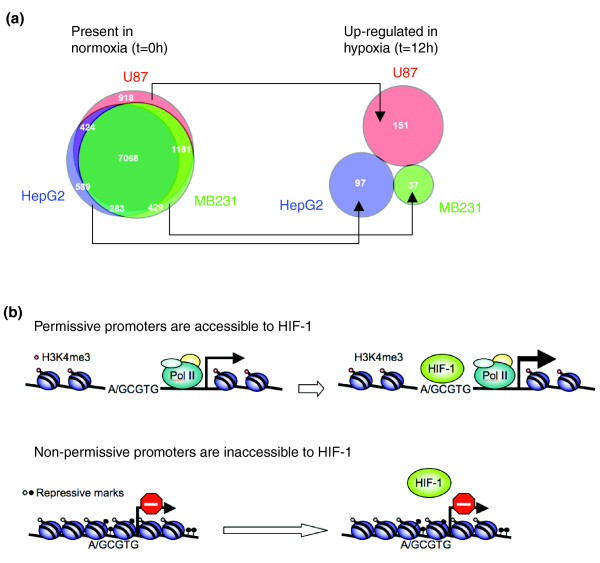
Basal gene expression predicts HIF-1 binding. **(a) **Proportional Venn diagram of genes with MAS5 present calls under normoxic conditions. Genes with basal mRNA production are largely overlapping among U87, HepG2, and MDA-MB231 cells (left panel). For the minority genes that were uniquely present in one cell line but not the other two, there was no overlap in their response to the onset of hypoxia (right panel). **(b) **Our results suggest that the repertoire of genes active in a cell (for example, through lineage specific transcription factors) defines the subset of genes that are permissive for binding and transactivation by stimulus-responsive transcription factors such as HIF-1. In this way, cell-type-specific differences in response to the same stimulus result, at least in part, from differences in basal gene expression profiles. Upon hypoxia, HIF-1 preferentially binds to active (permissive) loci, as indicated by the presence of H3K4 me3, RNA Pol II, or active mRNA production.

When comparing the gene expression profiles of the three cell lines, we found that genes with present expression under basal conditions largely overlapped (Figure 5a, 'Present in normoxia'). For the minority genes that were uniquely expressed in one cell line but not the other two, there was absolutely no overlap in their response to the onset of hypoxia (Figure 5a, 'Up-regulated in hypoxia'). Together, these results suggest that cell-type-specific gene expression profiles dictate the subset of genes that are permissive for regulation by stimulus-responsive transcription factors such as HIF-1 (Figure 5b). In the case of hypoxia-responsive genes, this concept applies not only to HIF-1 (Figures 1, 2 and 3), but also to secondary and HIF-independent modulators of gene expression (Figures 4 and 5).

## Discussion

We demonstrate here that when cells are acutely exposed to hypoxia, newly stabilized HIF-1 preferentially binds to loci that are already transcriptionally active under normal growth conditions, as indicated by the presence of RNA Pol II, H3K4 me3 modification, and basal mRNA production. This is similar to the findings for Myc, which preferentially binds to sites with H3K4 and H3K79 methylation and histone H3 acetylation [30,31]. Although Myc and HIF-1 binding to DNA can be precluded by methylation of their cognate DNA binding sequences [10,12,13,32], it has been shown that the presence of CpG methylation can only account for a minority of Myc binding exclusion and that Myc binding has a stronger dependence on H3K4 me3 [30].

It is likely that preferential binding to transcriptionally active loci is not specific to HIF-1 and Myc, but rather is generalizable to a variety of acutely activated transcription factors. For example, CREB binding is highly tissue-specific, and binding is apparent at genes that are transcriptionally active but not at promoters of genes that are not expressed [33]. Therefore, the panoply of epigenetic modifications that signify 'permissiveness' for binding is incompletely understood, but theses studies all support a model in which acutely activated transcription factors preferentially bind to loci that are already transcriptionally active. Of note, since normoxic cells have low levels of HIF-1, it is possible that low levels of HIF-1 binding actually help maintain the permissive state of certain high affinity sites under normoxic conditions. Furthermore, hypoxia under physiological or pathophysiological conditions can be acute, chronic or episodic. It is likely that with prolonged hypoxia, additional binding sites - for example, lower affinity biding sites - become occupied by HIF-1.

Comparing two different cell types, U87 and HepG2 cells, concordant HIF-1 binding was observed at many loci. Where binding was found to be discordant, in most cases there were differences in the epigenetic marking and basal transcriptional activity of the locus. These results suggest that the basal gene expression profile of cells may dictate the subset of loci to which stimuli-responsive transcription factors can bind. This concept is supported by a genome-wide analysis of FoxA1 binding in which cell-type-specific H3K4 me2 modification of enhancers predicted binding of FoxA1 [8]. Also, STAT1 has been found to preferentially bind to H3K4 me1-modified enhancers, thereby determining cell-type-specific differences in target gene responsiveness to interferon-γ treatment [34]. Together, these results suggest that the repertoire of genes active in a cell (for example, through lineage-specific transcription factors) defines the subset of genes that are permissive for binding and transactivation by stimulus-responsive transcription factors. In this way, cell-type-specific differences in response to the same stimulus results, at least in part, from differences in basal gene expression profiles.

## Conclusions

Many transcription factors are acutely activated in a stimulus-responsive manner. Although the canonical binding sequence is the same in all cells, there are often vast differences between different cell types in the loci bound by the same transcription factor. With acute activation of HIF-1, we have found that the transcription factor preferentially binds to loci that are already transcriptionally active under basal growth conditions. In two different cell lines, almost all HIF-1 binding sites are characterized by the presence of RNA Pol II, histone H3 methylation at lysine 4, or basal mRNA production. In the two cell lines, differences in basal transcriptional activity predicted differences in HIF-1 binding. These data, along with existing studies for Myc, STAT1, CREB and FoxA1, suggest that when transcription factors are acutely activated, they initially bind to loci that are already active. Therefore, differences in basal gene expression (for example, through lineage specific transcription factors) may largely dictate the subset of genes available for binding by stimulus-responsive factors, and may be the basis for cell type specificity in the pattern of binding by many transcription factors.

## Materials and methods

### Chromatin immunoprecipitation

ChIPs were performed as previously described [2,5] with minor modifications. Briefly, U87 cells were cultured for 4 h under normoxic or hypoxic (0.5% O_2_) conditions. Cells were fixed with 1% formaldehyde (37°C, 10 minutes) and lysed with 0.5% SDS lysis buffer. Chromatin was then sonicated to 500- to 1,000-bp fragments and immunoprecipitation carried out with HIF-1α pAb (NB 100-134 - Novus Biologicals, Littleton, CO, USA). RNA Pol II, H3K4 me3, and H3K27 me3 ChIPs were carried out using normoxic U87 or HepG2 cell samples with RNA Pol II mAb (ab5408 - Abcam, Cambridge, MA, USA), H3K4 me3 pAb (ab8580 - Abcam), and H3K27 me3 pAb (07-449 - Millipore, Billerica, MA, USA). DNA amplification, fragmentation, labeling, and hybridization were performed as previously described [5]. All ChIP samples were hybridized onto Affymetrix Human Promoter Tiling Array 1.0R.

### Identification of ChIP hits

The MAT algorithm [22] was used to identify regions enriched by ChIP-chip (ChIP hits). For the U87 HIF-1 ChIP, the triplicate hypoxic U87 HIF-1 ChIP samples were compared directly to triplicate normoxic samples. MAT was run with the parameters: bandwidth = 200, maximum gap = 400, minimum probes = 10, and *P*-value cutoff = 1 × 10^-5^. For H3K4 me3, H3K27 me3, and RNA Pol II ChIPs, normoxic ChIP samples were compared to matched input samples; the MAT parameters were increased to account for broader peaks (bandwidth = 500, maximum gap = 400, minimum probes = 20, and *P*-value cutoff = 1 × 10^-5^). The MAT library and mapping files were based on the March 2006 Human Genome Assembly (HG18). Hits flagged by MAT as mapping to repeat regions were excluded from consideration in all cases.

### Quantitative real-time PCR validation of ChIP hits

Primers were designed to span the peak intensity for each region of interest and against two negative control regions. For HIF-1, H3K4 me3, and RNA Pol II ChIPs, 5 kb and 10 kb upstream of the vascular endothelial growth factor (*VEGF*) gene were used as negative control regions. For H3K27 me3 ChIPs, promoter regions of the glyceraldehyde 3-phosphate dehydrogenase (*GAPDH*) and aldolase A (*ALDOA*) genes were used as negative control regions. Fold enrichment was assessed by performing qPCR for the target region on samples taken before (Input) and after ChIP (ChIP) and calculated from the critical threshold cycles (Ct) as: Fold enrichment = Target region ratio [2Δ^Ct(Ct ChIP-Ct Input)^]/Control region ratio [2Δ^Ct(Ct ChIP-Ct Input)^]. Specific binding was defined as a greater than twofold enrichment compared to matched control samples.

### Expression microarray

HepG2 hepatoma, U87 glioma, and MDA-MB231 breast cancer cells were collected under normoxic conditions (approximately 19% O_2_, 0 h) and after 4, 8 and 12 h of hypoxia treatment (0.5% O_2_). For each cell line, three replicates of total RNA at each time point were prepared using Trizol and submitted to the DFCI Microarray Core for labeling, hybridization to Affymetrix HG-U133Plus2 oligonucleotide arrays and image scanning. We used *GcRMA *module on Bioconductor with an updated custom CDF file [35] to normalize the microarrays. The MAS5 algorithm was used to make present/absent calls. LIMMA was used to identify probe sets whose expression levels were significantly changed after 4, 8, or 12 h of hypoxia relative to the normoxic signal. The MAS5 present/absent calls were assigned values of absent = 0, marginal = 0.5, or present = 1. For each probe set, the sum of triplicate samples was partitioned into 'present' if sum ≥ 2, and 'absent' if sum <2.

### Gene set enrichment analysis

We created gene sets containing all genes that could be associated with a ChIP hit. These sets were added to a file of gene sets (c5.mf.v2.5.symbols.gmt) downloaded from the GSEA website at the Broad Institute [36]. We used the command line version of GSEA2.0 with gene set permutation to derive significance, signal-to-noise as the distance metric and maximum expression to collapse probe sets to genes.

### Linking ChIP hits to RefSeq genes and expression profile

ChIP hits were associated with RefSeq genes from the University of California Santa Cruz (UCSC) RefGene table for HG18 based on chromosomal position. For analyzing the relationship between H3K4 me3, RNA Pol II and HIF-1 binding, only hits for which the binding peaks are ± 5 kb from the TSS of a gene were associated with the gene in order to minimize ambiguous assignment.

### Data access

The raw data are available from the NCBI Gene Expresion Omnibus database with accession number [GEO:GSE16347] for HepG2 HIF-1α ChIP-chip data and [GEO:GSE18505] for all other microarray and ChIP-chip data.

## Abbreviations

ChIP: chromatin immunoprecipitation; ChIP-chip: ChIP coupled with analysis on tiled microarrays; GSEA: gene set enrichment analysis; H3K4 me3: histone H3 trimethyl-lysine 4; H3K27 me3: histone H3 trimethyl-lysine 27; HIF-1: Hypoxia-inducible factor-1; MAT: Model-based Analysis of Tiling-array; qPCR: quantitative PCR; RNA Pol II: RNA polymerase II; TSS: transcriptional start site.

## Authors' contributions

XX and ALK designed the experiments. XX performed the experiments and analyzed the data. XX and ALK wrote the paper.

## Additional data files

The following additional data are available with the online version of this paper: a table listing all HIF-1 bound regions identified by ChIP-chip in U87 cells (Additional data file 1); GSEA analysis of HIF-1 binding and hypoxia-induced gene expression (Additional data file 2); HIF-1 binding associations with RNA Pol II and H3K4 me3 (Additional data file 3); a table listing all common HIF-1-bound loci identified by ChIP-chip in U87 cells and HepG2 cells (Additional data file 4).

## Supplementary Material

Additional data file 1 All HIF-1 bound regions identified by ChIP-chip in U87 cells. Click here for file

Additional data file 2 GSEA analysis of HIF-1 binding and hypoxia-induced gene expression. Click here for file

Additional data file 3 HIF-1 binding associations with RNA Pol II and H3K4 me3. Click here for file

Additional data file 4 All common HIF-1-bound loci identified by ChIP-chip in U87 cells and HepG2 cells. Click here for file
