# Supplementary material for: Preferential binding of HIF-1 to transcriptionally active loci determines cell-type specific response to hypoxia
Source: Genome Biol. 2009 Oct 14;10(10):R113. doi: 10.1186/gb-2009-10-10-r113 (PMC2784328; doi:10.1186/gb-2009-10-10-r113)
Supplement: Additional data file 1 — All HIF-1 bound regions identified by ChIP-chip in U87 cells. [file gb-2009-10-10-r113-S1.PDF]

Supplemental Table 1. HIF-1 Binding Regions identified by ChIP-chip in U87 cells (p-value<1e-8, promoter array)

	Position (hg18)		-10log10	TSS			U87 gene expr.				HepG2 gene expr.			
Chr	Start	End	_Pvalue	distance	Location	Gene	0	4	8	12	0	4	8	12
chr1	8861013	8862094	167.18	-250	Promoter	ENO1	P				P			
chr1	9049743	9050243	100.58	2262	Promoter	SLC2A5	A				A			
chr1	9925458	9926433	124.8	-473	Promoter	LZIC	P				P			
chr1	9925458	9926433	124.8	-186	Promoter	NMNAT1	P				P			
chr1	11912340	11912907	163.85	-3521	Promoter	KIAA2013	NA							
chr1	11912340	11912907	163.85	-4744	Promoter	PLOD1	P				P			
chr1	16275905	16276539	97.11	-3468	Promoter	FAM131C	A				A			
chr1	24024270	24024740	96.92	8	Promoter	HMGCL	P				A			
chr1	28434776	28435719	159.28	-3156	Promoter	DNAJC8	P				P			
chr1	28434776	28435719	159.28	88	Promoter	ATPIF1	P				P			
chr1	33274449	33275414	200.19	-123	Promoter	AK2	P				P			
chr1	36327666	36328368	107.29	850	Promoter	ADPRHL2	P				P			
chr1	36327666	36328368	107.29	5660	3prime	TEKT2	A				A			
chr1	40121448	40121979	151.25	-3	Promoter	TRIT1	P				P			
chr1	40399174	40400003	104.32	-205	Promoter	RLF	P				P			
chr1	40612367	40613415	115.25	649	Promoter	SMAP2	P				P			
chr1	43200462	43201670	221.43	-3540	Promoter	SLC2A1	P				P			
chr1	45013356	45014139	101.53	-121	Promoter	RPS8	A				A			
chr1	54076105	54077083	161.91	121	Promoter	TMEM48	P				P			
chr1	59022870	59023528	95.43	-891	Promoter	JUN	P				A			
chr1	72521523	72522527	194.98	-1141	Promoter	NEGR1	P				A			
chr1	109890091	109891231	112.16	-2034	Promoter	GNAI3	P				P			
chr1	109890091	109891231	112.16	6658	3prime	GPR61	A				A			
chr1	112810215	112810749	86.56	-1063	Promoter	WNT2B	A				A			
chr1	114156240	114157293	256.92	-121	Promoter	RSBN1	P				P			
chr1	114156240	114157293	256.92	59143	3prime	PTPN22	P				A			
chr1	144218571	144219341	108.81	-8680	Promoter	PEX11B	P				P			
chr1	144218571	144219341	108.81	65	Promoter	RBM8A	P				P			
chr1	145181168	145182131	173.1	689	Promoter	CHD1L	P				P			
chr1	148305582	148306400	113.22	-21	Promoter	VPS45	P				A			
chr1	148520956	148522072	106.68	-528	Promoter	C1orf51	P				A			
chr1	148520956	148522072	106.68	9503	3prime	C1orf54	P				A			
chr1	159664356	159665170	212.32	>50kb	long									
chr1	165455465	165456526	130.49	-755	Promoter	POU2F1	A				P			
chr1	184610941	184611672	98.63	-297	Promoter	C1orf27	P				P			
chr1	184610941	184611672	98.63	-246	Promoter	TPR	P				P			
chr1	200027134	200028140	222.92	143578	intragenic	NAV1	P				A			
chr1	201046714	201047959	221.87	-3091	Promoter	JARID1B	P				P			
chr1	226740812	226741628	110.61	-29018	within50k	HIST3H2A	NA							
chr1	226740812	226741628	110.61	28771	within50k	HIST3H2BB	NA							
chr1	234754094	234754937	203.43	854	Promoter	LGALS8	P				P			
chr2	665934	667028	127.38	810	Promoter	TMEM18	P				P			
chr2	10178036	10178796	92.21	-1815	Promoter	RRM2	P				P			
chr2	15618061	15619044	210.11	108	Promoter	NAG	P				P			
chr2	37164462	37165169	100.73	-173	Promoter	CCDC75	A				A			
chr2	37164462	37165169	100.73	65	Promoter	HEATR5B	P				P			
chr2	39204502	39205694	219.18	-3866	Promoter	SOS1	A				P			
chr2	54640252	54641046	103.2	1722	Promoter	SPTBN1	P				P			
chr2	55697615	55698826	131.45	134	Promoter	SMEK2	P				P			
chr2	63668800	63669777	153.44	67	Promoter	LOC51057	P				P			
chr2	63668800	63669777	153.44	-337	Promoter	MDH1	P				P			
chr2	63922892	63924070	99.92	769	Promoter	UGP2	P				P			
chr2	69821688	69822337	100.11	-692	Promoter	ANXA4	P				P			
chr2	70166971	70168244	156.6	-349	Promoter	PCBP1	P				P			
chr2	73911210	73912139	194.19	2134	Promoter	STAMBP	P				P			
chr2	74501830	74502899	155.56	21	Promoter	WDR54	P				P			
chr2	85675895	85676670	99.67	-1	Promoter	RNF181	P				P			
chr2	85675895	85676670	99.67	11305	3prime	VAMP5	P				A			
chr2	85675895	85676670	99.67	6947	3prime	TMEM150	A				P			
chr2	86520960	86521968	237.98	-415	Promoter	JMJD1A	P				P			
chr2	88136279	88136913	81.64	-272	Promoter	KRCC1	P				P			
chr2	95194986	95196010	117.59	-6530	Promoter	ZNF514	P				P			
chr2	95194986	95196010	117.59	611	Promoter	ZNF2	P				P			
chr2	97645363	97646202	102.99	1046	Promoter	ACTR1B	P				P			
chr2	118561641	118562716	359.79	-296	Promoter	INSIG2	P				P			
chr2	136458405	136459891	170.14	741	Promoter	DARS	P				P			
chr2	176573989	176575014	218.25	737	Promoter	KIAA1715	P				P			
chr2	202838506	202839291	89.24	95	Promoter	NOP5/NOP58	P				P			
chr2	206848313	206848982	105.91	819	Promoter	ZDBF2	P				A			
chr2	207337757	207338758	94.4	-40	Promoter	MDH1B	A				A			
chr2	207337757	207338758	94.4	-1275	Promoter	FASTKD2	P				P			
chr2	219141067	219141849	98.96	-144	Promoter	USP37	P				P			

chr2	219141067	219141849	98.96	-449	Promoter	RQCD1	P	P
chr2	219791199	219792310	157.44	35	Promoter	ABCB6	A	P
chr2	219791199	219792310	157.44	10724	3prime	ATG9A	P	P
chr2	219802754	219803786	132.16	438	Promoter	ANKZF1	A	A
chr2	219802754	219803786	132.16	-686	Promoter	ATG9A	P	P
chr2	219852430	219853269	185.56	560	Promoter	DNAJB2	P	P
chr2	220149591	220150244	96.04	4606	3prime	INHA	A	P
chr2	220149591	220150244	96.04	-5548	Promoter	OBSL1	P	P
chr2	223620131	223620879	82.34	-4653	Promoter	KCNE4	P	A
chr2	238173478	238174080	101.24	-9251	Promoter	RAB17	A	P
chr3	10131863	10132613	279.18	-192	Promoter	C3orf10	P	P
chr3	10131863	10132613	279.18	-7225	Promoter	C3orf24	A	A
chr3	14034891	14035629	117.3	>50kb	long			
chr3	38180918	38182012	95.64	-336	Promoter	OXSR1	P	P
chr3	48569043	48570033	466.58	-375	Promoter	PFKFB4	P	A
chr3	49760160	49761169	112.9	38344	intragenic	IHPK1	P	P
chr3	49798784	49799430	95.64	-164	Promoter	IHPK1	P	P
chr3	50349150	50350178	80.66	84	Promoter	RASSF1	P	A
chr3	50349150	50350178	80.66	-9143	Promoter	TUSC2	P	P
chr3	57516894	57517981	81.8	641	Promoter	PDE12	P	P
chr3	64645888	64646849	257.38	2257	Promoter	ADAMTS9	A	A
chr3	81892351	81894167	193.51	806	Promoter	GBE1	P	P
chr3	88190702	88191442	135.91	-174	Promoter	CGGBP1	P	P
chr3	98965406	98966451	222.22	-292	Promoter	ARL6	P	P
chr3	101693391	101694816	189.52	185	Promoter	TMEM45A	P	P
chr3	114715871	114716897	255.75	350	Promoter	CCDC52	P	P
chr3	123585016	123586104	281.37	-149	Promoter	C3orf28	P	P
chr3	123585016	123586104	281.37	-799	Promoter	CCDC58	P	A
chr3	127905151	127905855	98.31	-371	Promoter	CHCHD6	P	P
chr3	133861330	133862107	141.67	-115	Promoter	UBA5	P	P
chr3	133861330	133862107	141.67	-49	Promoter	ACAD11	P	P
chr3	147361508	147362505	138.72	-40	Promoter	PLOD2	P	P
chr3	150425395	150426257	159.46	-3271	Promoter	CP	A	P
chr3	154360698	154361420	106.31	-1774	Promoter	RAP2B	P	P
chr3	158027053	158027624	81.77	585	Promoter	LEKR1	NA	
chr3	185450691	185451186	82.64	-1499	Promoter	ALG3	P	P
chr3	185450691	185451186	82.64	801	Promoter	ECE2	A	A
chr3	185563524	185564362	163.71	99	Promoter	POLR2H	P	P
chr3	185563524	185564362	163.71	-1901	Promoter	CLCN2	A	A
chr3	196473223	196474157	116.46	-386	Promoter	C3orf21	A	A
chr3	199170890	199171873	173.57	-127	Promoter	IQCG	P	A
chr3	199170890	199171873	173.57	-69	Promoter	LMLN	A	A
chr4	7120323	7121016	92.45	-43	Promoter	GRPEL1	P	P
chr4	37364729	37365479	121.72	-814	Promoter	RELL1	A	A
chr4	68249159	68249978	98.18	-208	Promoter	UBA6	P	P
chr4	72315343	72316384	336.66	44023	intragenic	SLC4A4	P	A
chr4	76868201	76868916	81.93	-252	Promoter	USO1	P	P
chr4	77446532	77447195	100.24	72	Promoter	STBD1	P	A
chr4	78298209	78299165	92.13	1425	Promoter	CCNG2	P	P
chr4	89963526	89964347	111.68	-428	Promoter	FAM13A1	P	P
chr4	99797294	99798282	160.79	920	Promoter	TSPAN5	P	A
chr4	120440857	120441746	585.09	161	Promoter	LOC401152	P	P
chr4	124537211	124538481	301.28	602	Promoter	SPRY1	P	P
chr4	140155496	140156452	142.93	-365	Promoter	CCRN4L	P	P
chr4	140436101	140436759	93.73	-102	Promoter	NDUFC1	P	P
chr4	140436101	140436759	93.73	-5616	Promoter	NARG1	P	P
chr4	154792638	154793470	133.63	186238	within50k	KIAA0922	A	A
chr4	154792638	154793470	133.63	-31705	within50k	TLR2	A	A
chr4	159350776	159351562	92.52	12	Promoter	TMEM144	P	P
chr4	159863671	159864270	157.91	9	Promoter	PPID	P	P
chr4	174529464	174530143	92.11	1113	Promoter	SAP30	P	P
chr4	184661566	184662327	85.98	-1324	Promoter	ING2	P	P
chr4	186554143	186554761	85.88	-342	Promoter	ANKRD37	P	A
chr4	186583860	186584923	245.69	-3188	Promoter	LOC441054	NA	
chr4	186583860	186584923	245.69	-288	Promoter	UFSP2	P	P
chr5	33475713	33476571	101.27	-546	Promoter	TARS	P	P
chr5	43100476	43101122	89.37	-24668	within50k	C5orf39	P	A
chr5	43228592	43229158	101.6	753	Promoter	MGC42105	P	A
chr5	58370751	58371623	87	546822	intragenic	PDE4D	P	P
chr5	68565932	68566721	137.89	2	Promoter	CDK7	P	P
chr5	76361608	76362350	116.53	-139	Promoter	AGGF1	P	P
chr5	90611548	90613506	284.65	>50kb	long			
chr5	95184962	95185852	146.82	-1002	Promoter	GLRX	P	P
chr5	99898582	99899081	89.59	-228	Promoter	TMEM157	P	P
chr5	108772406	108773197	107.67	851	Promoter	PJA2	P	P
chr5	110455560	110456793	83.32	285	Promoter	WDR36	P	P
chr5	111121341	111122109	95.22	-833	Promoter	C5orf13	P	P

chr5	118351783	118352512	123.82	-53	Promoter	DTWD2	NA	
chr5	118433951	118434897	98.86	-525	Promoter	DMXL1	P	P
chr5	121439976	121440586	81.01	1628	Promoter	LOX	P	P
chr5	121441545	121442514	242.73	-177	Promoter	LOX	P	P
chr5	130998775	130999602	113.25	-411	Promoter	RAPGEF6	P	P
chr5	133368407	133369213	107.1	-338	Promoter	VDAC1	P	P
chr5	137938931	137939811	182.87	-428	Promoter	HSPA9	P	P
chr5	138704564	138705517	141.4	-605	Promoter	PAIP2	P	P
chr5	139923856	139924750	166.4	-39	Promoter	APBB3	P	A
chr5	139923856	139924750	166.4	-191	Promoter	SLC35A4	P	A
chr5	139923856	139924750	166.4	-6550	Promoter	SRA1	P	P
chr5	145194489	145195016	103.92	330	Promoter	PRELID2	P	A
chr5	158566999	158567979	159.41	-49	Promoter	RNF145	P	P
chr5	162819289	162820303	139.92	-145	Promoter	NUDCD2	P	P
chr5	162819289	162820303	139.92	-374	Promoter	HMMR	P	P
chr5	172688241	172689351	101.64	625	Promoter	STC2	P	P
chr5	176875812	176876982	138.64	175	Promoter	DDX41	P	P
chr5	176875812	176876982	138.64	37746	3prime	FLJ10404	P	A
chr5	176875812	176876982	138.64	-6939	Promoter	DOK3	A	A
chr5	179091718	179092676	114.31	-70	Promoter	MAML1	P	P
chr5	179091718	179092676	114.31	33851	3prime	CANX	P	P
chr6	5952029	5952792	99.62	282	Promoter	NRN1	P	A
chr6	5953230	5953699	101.38	-810	Promoter	NRN1	P	A
chr6	29867795	29868692	155.53	-34482	within50k	HLA-G	P	P
chr6	43844532	43845466	213.3	-982	Promoter	VEGFA	P	P
chr6	53623976	53625236	240.51	13920	intragenic	KLHL31	A	A
chr6	87921242	87922686	198.12	-79	Promoter	ZNF292	P	P
chr6	89847972	89848681	98.49	1041	Promoter	PNRC1	P	P
chr6	99979589	99981099	87.3	10	Promoter	SFRS18	P	P
chr6	114283910	114284850	98.89	-927	Promoter	MARCKS	P	P
chr6	126143221	126144298	150.84	-9942	Promoter	NCOA7	P	P
chr6	143813320	143814069	92.61	-115	Promoter	ADAT2	P	P
chr6	143813320	143814069	92.61	-160	Promoter	PEX3	P	P
chr7	12692347	12693081	103.53	-298	Promoter	ARL4A	N/A	
chr7	25131191	25131919	91.7	9	Promoter	CYCS	P	P
chr7	29994338	29995228	137.75	1074	Promoter	SCRN1	P	P
chr7	30034886	30035616	104.33	518	Promoter	PLEKHA8	A	A
chr7	30034886	30035616	104.33	-2537	Promoter	FKBP14	P	P
chr7	35806386	35807505	376.27	-186	Promoter	7-Sep	A	A
chr7	47546364	47547083	80.35	-959	Promoter	TNS3	P	P
chr7	54793986	54795070	341.53	-145	Promoter	SEC61G	P	P
chr7	75826495	75827138	83.42	-565	Promoter	YWHAG	P	P
chr7	100547127	100547788	94.12	-9556	Promoter	SERPINE1	P	P
chr7	100556470	100557242	85.62	-219	Promoter	SERPINE1	P	P
chr7	100647583	100648319	143.59	-197	Promoter	PLOD3	P	P
chr7	100647583	100648319	143.59	224	Promoter	ZNHIT1	P	P
chr7	104440016	104441154	219.4	-1259	Promoter	MLL5	P	P
chr7	107953587	107954409	91.41	-217	Promoter	PNPLA8	P	P
chr7	107996793	107997391	87.83	-502	Promoter	DNAJB9	P	P
chr7	107996793	107997391	87.83	44	Promoter	THAP5	A	A
chr7	127078944	127079687	94.4	-130	Promoter	SND1	P	P
chr7	127882485	127883839	426.73	-39	Promoter	HIG2	P	P
chr7	133652004	133652628	81.52	-73	Promoter	SLC35B4	P	P
chr7	142278668	142279166	96.08	15997	intragenic	EPHB6	A	A
chr7	142278668	142279166	96.08	14689	3prime	TRPV6	A	A
chr7	148454845	148455473	90.3	-873	Promoter	ZNF425	A	A
chr7	148454845	148455473	90.3	744	Promoter	ZNF398	P	P
chr7	154719237	154720064	116.91	-777	Promoter	INSIG1	P	A
chr8	22510985	22512058	87.72	17628	3prime	PDLIM2	P	P
chr8	22510985	22512058	87.72	-1510	Promoter	C8orf58	P	A
chr8	22510985	22512058	87.72	-6645	Promoter	KIAA1967	P	P
chr8	23077043	23077804	95.6	41	Promoter	TNFRSF10D	P	P
chr8	23767252	23768051	142.83	576	Promoter	STC1	P	A
chr8	26295881	26296666	159.97	-233	Promoter	BNIP3L	P	P
chr8	26297044	26297728	138.6	900	Promoter	BNIP3L	P	P
chr8	35502389	35503029	82.99	-18776	within50k	UNC5D	NA	
chr8	49665118	49665758	99.9	>50kb	long			
chr8	52933861	52934574	127.17	2201	Promoter	PCMTD1	P	P
chr8	62789506	62790565	230.22	-312	Promoter	ASPH	P	P
chr8	79590208	79591083	112.66	-213	Promoter	PKIA	P	A
chr8	104495846	104496628	88.29	101	Promoter	WDSOF1	P	P
chr8	104495846	104496628	88.29	229	Promoter	SLC25A32	P	P
chr9	6747430	6747997	86.49	60	Promoter	JMJD2C	A	P
chr9	15412329	15413093	101.74	-161	Promoter	SNAPC3	P	P
chr9	19039967	19040859	80.45	1060	Promoter	RRAGA	P	P
chr9	19039967	19040859	80.45	52471	3prime	FAM29A	P	P
chr9	34446942	34447827	101.65	1059	Promoter	C9orf25	P	P

chr9	34446942	34447827	101.65	-1301	Promoter	DNAI1	A				A			
chr9	35663329	35664342	170.64	-83	Promoter	CA9	P				P			
chr9	35663329	35664342	170.64	-8553	Promoter	C9orf100	P				A			
chr9	85784015	85784713	92.93	629	Promoter	HNRNPK	P				P			
chr9	85784015	85784713	92.93	-1081	Promoter	RMI1	P				P			
chr9	99434815	99435824	98.07	536	Promoter	C9orf97	P				P			
chr9	99434815	99435824	98.07	-278	Promoter	NCBP1	P				P			
chr9	99724027	99724707	95.79	322	Promoter	C9orf156	P				P			
chr9	102228549	102229190	142.08	-664	Promoter	C9orf30	P				P			
chr9	102231091	102231816	181.11	2002	Promoter	C9orf30	P				P			
chr9	105895965	105896824	153.12	33	Promoter	SMC2	P				P			
chr9	109076828	109077433	94.78	-8289	Promoter	RAD23B	P				P			
chr9	110638758	110639254	101.22	-25417	within50k	ACTL7A	A				A			
chr9	110638758	110639254	101.22	19025	within50k	ACTL7B	A				A			
chr9	110638758	110639254	101.22	97423	within50k	IKBKAP	P				P			
chr9	115022950	115023822	110.19	84	Promoter	FKBP15	P				P			
chr9	115022950	115023822	110.19	-310	Promoter	SLC31A1	P				P			
chr9	125732303	125732808	81.47	-313	Promoter	DENND1A	P				P			
chr9	126743016	126743805	98.19	-241	Promoter	GOLGA1	P				P			
chr9	129380924	129381528	145.96	-47	Promoter	FAM129B	P				P			
chr9	130172723	130173940	126.08	-357	Promoter	URM1	P				P			
chr9	134534771	134535336	92.77	549	Promoter	DDX31	A				A			
chr9	134534771	134535336	92.77	-488	Promoter	GTF3C4	P				P			
chr9	138861812	138862679	143.28	3592	3prime	LOC389813	NA							
chr9	138861812	138862679	143.28	-1087	Promoter	PHPT1	P				P			
chr9	138861812	138862679	143.28	-4360	Promoter	MAMDC4	A				A			
chr10	6283526	6284159	135.69	-1135	Promoter	PFKFB3	P				A			
chr10	6286550	6287244	85.48	2095	Promoter	PFKFB3	P				A			
chr10	21855376	21856069	83.04	-7321	Promoter	MLLT10	P				P			
chr10	21855376	21856069	83.04	-1169	Promoter	C10orf140	P				A			
chr10	25345402	25346060	86.27	-616	Promoter	C10orf63	A				A			
chr10	25345402	25346060	86.27	139	Promoter	THNSL1	P				P			
chr10	27429000	27429851	115.89	42	Promoter	ANKRD26	P				A			
chr10	27569033	27569679	80.07	343	Promoter	ACBD5	P				P			
chr10	44797166	44798179	225.82	-3362	Promoter	C10orf10	P				A			
chr10	44797166	44798179	225.82	22474	intragenic	RASSF4	P				P			
chr10	53129018	53129766	93.84	625205	intragenic	PRKG1	P				A			
chr10	53129018	53129766	93.84	-83	Promoter	CSTF2T	P				P			
chr10	59695674	59696702	161.21	1513	Promoter	IPMK	A				A			
chr10	59695674	59696702	161.21	-2713	Promoter	CISD1	P				P			
chr10	70330615	70331431	134.56	38	Promoter	DDX50	P				P			
chr10	70385296	70386092	125.05	-163	Promoter	DDX21	P				P			
chr10	73703018	73704030	184.92	-201	Promoter	DDIT4	P				P			
chr10	74526279	74527196	150.87	-175	Promoter	P4HA1	P				P			
chr10	75173592	75174345	100.72	-159	Promoter	SEC24C	P				P			
chr10	75202147	75203309	209.61	565	Promoter	FUT11	P				A			
chr10	75202147	75203309	209.61	28482	3prime	SEC24C	P				P			
chr10	75202147	75203309	209.61	-9194	Promoter	CHCHD1	P				P			
chr10	93382226	93383743	277.82	-253	Promoter	PPP1R3C	P				P			
chr10	99175696	99176332	161.45	-108	Promoter	PGAM1	NA							
chr10	99883675	99884645	114.66	-114	Promoter	C10orf28	P				P			
chr10	102095997	102096633	189.9	-552	Promoter	SCD	P				P			
chr10	103337590	103338236	137.05	-91	Promoter	RP11-529I10.4	P				P			
chr10	103337590	103338236	137.05	-24	Promoter	POLL	A				A			
chr10	111756234	111757252	195.49	-922	Promoter	ADD3	P				P			
chr10	121641632	121642388	111.6	-162	Promoter	SEC23IP	P				P			
chr11	6904076	6905032	170.23	405	Promoter	ZNF215	A				A			
chr11	9242493	9243096	81.54	701	Promoter	RAB6IP1	P				P			
chr11	11599110	11600008	99.06	504	Promoter	GALNTL4	P				A			
chr11	14498163	14499232	167.76	-114	Promoter	PSMA1	P				P			
chr11	17991390	17992311	167.72	-625	Promoter	SERGEF	P				P			
chr11	18300152	18300812	80.05	-117	Promoter	HPS5	P				P			
chr11	18300152	18300812	80.05	-304	Promoter	GTF2H1	P				P			
chr11	18372355	18373091	113.43	74	Promoter	LDHA	P				P			
chr11	27340552	27341599	148.48	292	Promoter	CCDC34	P				P			
chr11	34083242	34084157	100.89	-53	Promoter	NAT10	P				P			
chr11	34083242	34084157	100.89	53866	3prime	CAPRIN1	P				P			
chr11	56864355	56864982	123.26	2139	Promoter	P2RX3	A				A			
chr11	56864355	56864982	123.26	-4736	Promoter	SSRP1	P				P			
chr11	56956702	56957262	120.5	-5313	Promoter	SLC43A3	P				A			
chr11	62226363	62226891	82.76	3752	intragenic	BSCL2	P				A			
chr11	62226363	62226891	82.76	-5097	Promoter	GNG3	A				A			
chr11	66951953	66952808	106.62	-161	Promoter	RPS6KB2	P				P			
chr11	66951953	66952808	106.62	12625	3prime	KIAA1394	A				A			
chr11	70841928	70842552	88.77	381	Promoter	NADSYN1	P				A			
chr11	70841928	70842552	88.77	-5120	Promoter	DHCR7	P				P			
chr11	71822675	71823516	106.89	56	Promoter	CLPB	P				A			

chr11	73559899	73560503	80.74	170	Promoter	PPME1	P				P			
chr11	73559899	73560503	80.74	-473	Promoter	C2CD3	A				A			
chr11	75832881	75833703	134.17	-407	Promoter	C11orf30	P				P			
chr11	82544680	82545324	141.78	-818	Promoter	PCF11	P				P			
chr11	107873448	107874046	83.39	641	Promoter	KDELC2	P				P			
chr12	2791823	2792502	167.08	131	Promoter	ITFG2	A				A			
chr12	6512353	6514190	206.25	-297	Promoter	GAPDH	P				P			
chr12	6512353	6514190	206.25	40062	3prime	NCAPD2	P				P			
chr12	6893385	6894045	90.43	-97	Promoter	ENO2	P				P			
chr12	6893385	6894045	90.43	9565	3prime	LRRC23	P				A			
chr12	10657128	10658015	205.28	-161	Promoter	MAGOHB	P				A			
chr12	12832941	12833545	153.65	3346	intragenic	APOLD1	P				A			
chr12	14814007	14815481	314.84	790	3prime	HIST4H4	NA							
chr12	14814007	14815481	314.84	-3994	Promoter	H2AFJ	P				P			
chr12	21545102	21545887	83.06	-637	Promoter	GOLT1B	P				P			
chr12	21545102	21545887	83.06	367	Promoter	RECQL	P				P			
chr12	24946294	24947349	116.78	46627	intragenic	BCAT1	P				P			
chr12	26168801	26169304	102.11	21	Promoter	BHLHB3	P				A			
chr12	26981607	26982257	94.08	547	Promoter	C12orf11	P				P			
chr12	26981607	26982257	94.08	-608	Promoter	FGFR1OP2	P				P			
chr12	55806997	55807844	165.71	-1030	Promoter	LRP1	P				P			
chr12	55845038	55846298	182.8	37214	intragenic	LRP1	P				P			
chr12	56452499	56453455	127.52	-5773	Promoter	CYP27B1	P				A			
chr12	56452499	56453455	127.52	-835	Promoter	METTL1	P				P			
chr12	56452499	56453455	127.52	-9786	Promoter	TSFM	P				P			
chr12	56452499	56453455	127.52	367	Promoter	FAM119B	P				P			
chr12	63131406	63132271	166.12	-325	Promoter	TBK1	P				P			
chr12	74191431	74192192	114.38	-184	Promoter	KRR1	P				P			
chr12	102847544	102848251	112.31	-491	Promoter	HSP90B1	P				P			
chr12	103848337	103849015	133.08	-2097	Promoter	SLC41A2	P				A			
chr12	109390318	109391126	127.17	87	Promoter	C12orf24	P				P			
chr12	109390318	109391126	127.17	-254	Promoter	GPN3	P				P			
chr12	119396714	119397469	264.52	-5093	Promoter	SFRS9	P				P			
chr12	119396714	119397469	264.52	4992	intragenic	DYNLL1	P				P			
chr12	120840196	120840979	152.1	-277	Promoter	WDR66	P				A			
chr12	120840196	120840979	152.1	29557	3prime	PSMD9	P				P			
chr12	121576897	121577908	176.14	127	Promoter	RSRC2	P				P			
chr12	121576897	121577908	176.14	-389	Promoter	KNTC1	P				P			
chr12	122030491	122030985	83.19	-97	Promoter	ARL6IP4	P				P			
chr12	122030491	122030985	83.19	-5030	Promoter	ABCB9	A				A			
chr12	122030491	122030985	83.19	5429	3prime	OGFOD2	P				P			
chr13	34947335	34948422	109.12	1024	Promoter	MAB21L1	P				A			
chr13	34947335	34948422	109.12	533309	intragenic	NBEA	P				P			
chr13	49407681	49408639	102.87	446	Promoter	C13orf1	P				P			
chr13	98948667	98949385	88.05	-2704	Promoter	TM9SF2	P				P			
chr13	110165339	110166434	141.55	329	Promoter	ING1	P				P			
chr13	110165339	110166434	141.55	-9224	Promoter	CARS2	P				P			
chr13	113112701	113114317	118.37	37512	within50k	ADPRHL1	A				A			
chr13	113112701	113114317	118.37	79078	within50k	DCUN1D2	P				P			
chr13	113112701	113114317	118.37	-47482	within50k	GRTP1	P				P			
chr14	44674267	44675125	129.63	-251	Promoter	FANCM	P				P			
chr14	44674267	44675125	129.63	-362	Promoter	FKBP3	P				P			
chr14	50631885	50632694	126.12	-543	Promoter	TRIM9	P				A			
chr14	56805040	56805788	88.14	-34	Promoter	EXOC5	P				P			
chr14	56805040	56805788	88.14	25	Promoter	C14orf108	P				P			
chr14	61083893	61084541	87.25	225978	intragenic	PRKCH	P				A			
chr14	63177626	63178594	154.06	-228	Promoter	WDR89	P				P			
chr14	64522898	64524128	424.73	177	Promoter	FNTB	P				P			
chr14	67231758	67232646	201.73	-6133	Promoter	RDH12	A				A			
chr14	67231758	67232646	201.73	41	Promoter	RDH11	P				P			
chr14	68330776	68331524	111.5	-1708	Promoter	ZFP36L1	P				P			
chr14	68330776	68331524	111.5	1697	Promoter	C14orf181	P				A			
chr14	74587588	74588162	101.37	153	Promoter	MLH3	P				P			
chr14	74587588	74588162	101.37	12654	3prime	ACYP1	P				A			
chr14	76993476	76994270	128.25	-287	Promoter	AHSA1	P				P			
chr14	76993476	76994270	128.25	-210	Promoter	C14orf133	P				A			
chr14	95810547	95811077	196.38	18484	within50k	BDKRB1	P				A			
chr14	95810547	95811077	196.38	69908	within50k	BDKRB2	P				A			
chr14	95810547	95811077	196.38	88636	within50k	ATG2B	P				P			
chr15	27349166	27349886	98.19	300804	intragenic	KIAA0574	NA							
chr15	27349166	27349886	98.19	-106	Promoter	NDNL2	P				P			
chr15	46957099	46958061	210.92	-20	Promoter	EID1	P				A			
chr15	46957099	46958061	210.92	85372	intragenic	SHC4	P				A			
chr15	50050962	50051636	96.2	40	Promoter	LEO1	P				P			
chr15	70455312	70456031	95.79	-135	Promoter	HEXA	P				P			
chr15	72968964	72969827	155.86	-77	Promoter	MPI	P				P			
chr15	73718606	73719773	172.4	-9233	Promoter	SNX33	P				P			

chr15	73718606	73719773	172.4	481	Promoter	IMP3	P	P
chr15	76378357	76379364	139.95	127	Promoter	WDR61	P	P
chr15	94671394	94671929	94.84	-3343	Promoter	NR2F2	P	P
chr15	99609349	99609966	91.01	5	Promoter	CHSY1	P	P
chr16	1816934	1818009	316.56	-303	Promoter	HAGH	P	P
chr16	1816934	1818009	316.56	269	Promoter	FAHD1	P	P
chr16	2000156	2000615	84.02	-9118	Promoter	NPW	A	A
chr16	2000156	2000615	84.02	-639	Promoter	ZNF598	A	A
chr16	8869013	8869812	110.46	384	Promoter	CARHSP1	P	P
chr16	29983762	29984851	196.81	-339	Promoter	ALDOA	P	P
chr16	31013246	31013896	282.99	93	Promoter	VKORC1	P	P
chr16	31013246	31013896	282.99	-6053	Promoter	POL3S	A	A
chr16	45475246	45475792	87.57	-315	Promoter	GPT2	P	P
chr16	49744623	49745548	137.91	-2381	Promoter	SALL1	P	P
chr16	65761291	65761993	98.86	-3661	Promoter	NOL3	P	A
chr16	65761291	65761993	98.86	6852	3prime	HSF4	A	A
chr16	88102510	88103237	93.74	686	Promoter	SPG7	P	P
chr17	582916	583585	106.62	637	Promoter	FAM57A	P	P
chr17	2186016	2186795	82.16	1057	Promoter	TSR1	P	P
chr17	2186016	2186795	82.16	-1184	Promoter	SGSM2	P	P
chr17	4581249	4582007	124.33	8324	3prime	CXCL16	A	P
chr17	4581249	4582007	124.33	177	Promoter	MED11	P	P
chr17	4581249	4582007	124.33	-8437	Promoter	ZMYND15	A	A
chr17	6495126	6495805	88.6	-258	Promoter	LOC388327	NA	
chr17	6495126	6495805	88.6	154	Promoter	MED31	P	P
chr17	7283766	7284491	92.58	719	Promoter	FGF11	A	A
chr17	7283766	7284491	92.58	-4998	Promoter	CHRNB1	A	A
chr17	7283766	7284491	92.58	4646	3prime	TMEM102	A	A
chr17	7416259	7417075	144.1	-6868	Promoter	CD68	P	A
chr17	7416259	7417075	144.1	-120	Promoter	EIF4A1	P	P
chr17	7416259	7417075	144.1	10618	3prime	SENP3	P	P
chr17	7759090	7759971	153.91	26624	3prime	CHD3	P	A
chr17	15789109	15789924	87.35	536	Promoter	ADORA2B	P	P
chr17	18026863	18027760	366.72	-483	Promoter	ALKBH5	P	P
chr17	18158727	18159496	105.36	-138	Promoter	TOP3A	P	P
chr17	18158727	18159496	105.36	-134	Promoter	SMCR8	P	P
chr17	19221553	19222442	97.43	298	Promoter	MAPK7	P	A
chr17	19821340	19822291	132.91	-22	Promoter	AKAP10	P	A
chr17	22644660	22645691	123.48	-126	Promoter	WSB1	P	P
chr17	35549494	35550152	98.89	-149	Promoter	CASC3	P	P
chr17	36587894	36588643	127.24	-228	Promoter	KRTAP4-2	A	A
chr17	39339376	39340093	85.89	953	Promoter	MPP2	P	A
chr17	39439074	39439840	125.24	1847	Promoter	NAGS	P	A
chr17	39439074	39439840	125.24	-2041	Promoter	PYY	A	A
chr17	43481089	43481728	131.7	786	Promoter	NFE2L1	P	P
chr17	46585393	46586479	171.97	-8	Promoter	NME1-NME2	P	P
chr17	46585393	46586479	171.97	-8	Promoter	NME1	P	P
chr17	53664855	53665378	102.65	-5716	Promoter	LPO	A	A
chr17	54997390	54998202	126.87	213	Promoter	DHX40	P	P
chr17	70960766	70961264	89.33	-3280	Promoter	KIAA0195	P	P
chr17	71861524	71862201	83.96	-329	Promoter	PRPSAP1	P	P
chr17	71891045	71891729	86.26	-1002	Promoter	SPHK1	P	P
chr17	72196005	72196810	153.36	22173	intragenic	MXRA7	P	P
chr17	72233361	72233921	98.36	838	Promoter	JMJD6	P	P
chr17	72233361	72233921	98.36	-906	Promoter	LOC124512	P	P
chr17	73883338	73883873	82.94	-2707	Promoter	PGS1	P	A
chr17	77261746	77262349	80.45	641	Promoter	HGS	P	P
chr17	77261746	77262349	80.45	-706	Promoter	ARL16	P	A
chr17	77411780	77412831	177.72	-414	Promoter	P4HB	P	P
chr17	78008892	78009920	89.91	-7188	Promoter	C17orf62	P	P
chr17	78008892	78009920	89.91	-177	Promoter	NARF	P	P
chr18	647701	648487	112.18	439	Promoter	TYMS	P	P
chr18	647701	648487	112.18	298	Promoter	C18orf56	P	P
chr18	9007309	9008118	86.63	>50kb	long			
chr18	13715980	13717075	346.96	-132	Promoter	RNMT	P	P
chr18	13715980	13717075	346.96	20	Promoter	C18orf19	P	P
chr18	17434204	17435086	92.11	-31	Promoter	ESCO1	P	P
chr18	19286570	19287558	162.05	277	Promoter	RIOK3	P	P
chr18	30327015	30327535	80.1	-51	Promoter	DTNA	P	A
chr18	69965729	69967478	278.37	-878	Promoter	FBXO15	P	A
chr18	69965729	69967478	278.37	82	Promoter	C18orf55	P	P
chr18	75539914	75541060	219.06	-256	Promoter	CTDP1	P	P
chr19	584122	584779	112.28	1	Promoter	POLRMT	P	P
chr19	584122	584779	112.28	-6358	Promoter	FGF22	A	A
chr19	3084013	3084619	155.13	-2812	Promoter	GNA15	A	A
chr19	3713320	3713982	89	-1007	Promoter	APBA3	P	A
chr19	3713320	3713982	89	16	Promoter	MRPL54	P	A

chr19	4919325	4920120	277.72	-485	Promoter	JMJD2B	P	P
chr19	5670735	5671951	228.47	-88	Promoter	LONP1	P	P
chr19	5670735	5671951	228.47	-423	Promoter	TMEM146	A	A
chr19	5741493	5742370	99.54	317	Promoter	DUS3L	P	A
chr19	5741493	5742370	99.54	-6263	Promoter	MGC24975	A	A
chr19	8332671	8333460	106.6	-1904	Promoter	ANGPTL4	P	A
chr19	10077562	10078611	158.46	181	Promoter	PPAN	P	P
chr19	10077562	10078611	158.46	-3720	Promoter	ANGPTL6	A	A
chr19	10077562	10078611	158.46	-5051	Promoter	P2RY11	A	A
chr19	10077562	10078611	158.46	75	Promoter	PPAN-P2RY11	A	A
chr19	12753954	12754569	115.27	-9073	Promoter	JUNB	P	A
chr19	12753954	12754569	115.27	-6802	Promoter	HOOK2	A	A
chr19	12928439	12929044	109.74	399	Promoter	GADD45GIP1	P	P
chr19	12996752	12997469	109.03	29525	intragenic	NFIX	P	A
chr19	16544182	16544877	174.98	-236	Promoter	SLC35E1	P	P
chr19	16544182	16544877	174.98	55586	3prime	MED26	P	P
chr19	17979428	17979961	105.43	-302	Promoter	ARRDC2	P	A
chr19	18529421	18529925	84.81	110	Promoter	C19orf50	P	P
chr19	39541621	39542714	171.48	-5858	Promoter	GPI	P	P
chr19	44031339	44032141	123.8	710	Promoter	HNRNPL	P	P
chr19	45028482	45029410	159.12	-145	Promoter	FBL	P	P
chr19	45623224	45623893	87.06	245	Promoter	SERTAD1	P	A
chr19	46594977	46595774	109.29	-122	Promoter	BCKDHA	P	P
chr19	46594977	46595774	109.29	-325	Promoter	EXOSC5	P	P
chr19	50274534	50275325	142.47	601	Promoter	GEMIN7	P	P
chr19	50274534	50275325	142.47	-3442	Promoter	ZNF342	A	P
chr19	50779485	50780302	160.78	-16	Promoter	OPA3	P	P
chr19	50887158	50887984	115.73	-224	Promoter	QPCTL	A	A
chr19	50887158	50887984	115.73	-265	Promoter	SNRPD2	P	P
chr19	52938316	52938987	91.65	-1953	Promoter	GLTSCR2	P	P
chr19	52938316	52938987	91.65	30254	3prime	EHD2	P	A
chr19	55071517	55072988	136.03	-9349	Promoter	PNKP	P	P
chr19	55071517	55072988	136.03	-661	Promoter	TBC1D17	P	P
chr19	55071517	55072988	136.03	411	Promoter	AKT1S1	P	P
chr19	62566054	62566864	104.96	-202	Promoter	ZNF547	A	A
chr19	63775928	63776978	312.56	48	Promoter	MZF1	P	A
chr20	33506531	33507196	90.56	250	Promoter	CEP250	P	P
chr20	33668381	33669391	215.9	1629	Promoter	SPAG4	P	P
chr20	33793320	33794080	134.64	-32	Promoter	RBM39	P	P
chr20	45379314	45379957	89.31	39206	intragenic	ZMYND8	P	P
chr20	54400721	54401545	144.86	71	Promoter	CSTF1	P	P
chr20	54400721	54401545	144.86	-426	Promoter	AURKA	P	P
chr21	25932822	25933609	116.37	-218	Promoter	JAM2	P	A
chr21	27140325	27141256	117.57	-1179	Promoter	ADAMTS1	P	A
chr21	29179133	29179918	87.11	-35	Promoter	N6AMT1	P	A
chr21	34209605	34210446	106.7	-105	Promoter	ATP5O	P	P
chr21	35181922	35182836	96.6	477	Promoter	RUNX1	P	A
chr21	35341726	35342425	96.12	1460	Promoter	RUNX1	P	A
chr22	16020056	16020755	82.46	-133	Promoter	CECR5	P	A
chr22	19600925	19601597	132.49	-470	Promoter	CRKL	P	P
chr22	22566197	22566946	115.76	-60	Promoter	MIF	P	P
chr22	29149013	29150577	377.19	-1742	Promoter	MTP18	P	P
chr22	29149013	29150577	377.19	27045	3prime	SEC14L2	P	A
chr22	29317336	29318199	119.88	173	Promoter	PES1	P	P
chr22	35254617	35255631	261.76	71	Promoter	EIF3D	P	P
chr22	36384103	36384760	87.23	-167	Promoter	PDXP	P	P
chr22	36384103	36384760	87.23	18886	3prime	SH3BP1	A	A
chr22	38045307	38046131	93.54	-87	Promoter	RPL3	P	P
chr22	42752544	42753775	310.77	1813	Promoter	PARVB	P	A
chr22	45110060	45110806	148.42	476	Promoter	TRMU	P	P
chr22	45110060	45110806	148.42	38962	3prime	GTSE1	P	P
chr22	45536619	45537461	142.72	-138	Promoter	TBC1D22A	P	P
chrX	21868032	21868817	104.55	-237	Promoter	SMS	NA	
chrX	47402866	47403476	110.25	-8211	Promoter	ELK1	P	P
chrX	47402866	47403476	110.25	302	Promoter	UXT	P	P
chrX	48219103	48219737	156.41	-5877	Promoter	SLC38A5	A	P
chrX	48219103	48219737	156.41	-106	Promoter	FTSJ1	P	P
chrX	53270879	53271572	97.24	126	Promoter	JARID1C	P	P
chrX	54401457	54402233	84.64	-603	Promoter	WNK3	A	A
chrX	74410114	74410949	124.57	-247	Promoter	UPRT	P	P
chrX	77245708	77246572	145.79	-297	Promoter	PGK1	P	P
chrX	133993804	133994664	156.99	264	Promoter	FAM127A	P	P

P: present
N/A: no probe on the microarray

>1 1 0.5 0 -0.5 -1 <-1

not significant (P>0.01)
