# Supplementary material for: Preferential binding of HIF-1 to transcriptionally active loci determines cell-type specific response to hypoxia
Source: Genome Biol. 2009 Oct 14;10(10):R113. doi: 10.1186/gb-2009-10-10-r113 (PMC2784328; doi:10.1186/gb-2009-10-10-r113)
Supplement: Additional data file 2 — GSEA analysis of HIF-1 binding and hypoxia-induced gene expression. [file gb-2009-10-10-r113-S2.PDF]

Xia_Additional2

A

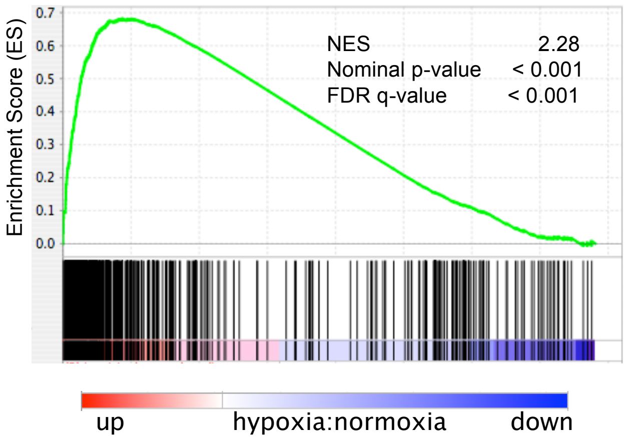

B

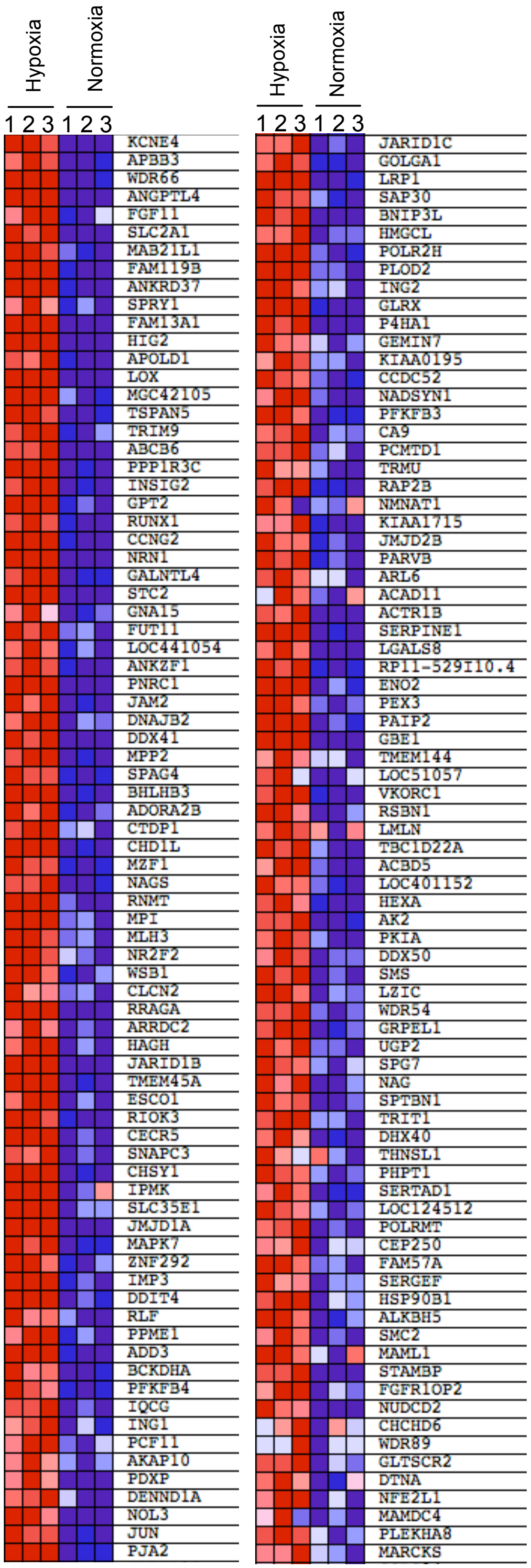

Figure S1. HIF-1 binding is associated with up-regulated expression under hypoxic conditions.

A. A gene set consisting of all HIF-1 ChIP-chip hits in U87 cells is highly enriched for genes whose expression are upregulated under hypoxic conditions.

B. Gene expression changes of the 158 leading edge genes from the GSEA analysis comparing hypoxic samples (0.5%O_2,12hr) to normoxic samples.
