# Supplementary material for: Preferential binding of HIF-1 to transcriptionally active loci determines cell-type specific response to hypoxia
Source: Genome Biol. 2009 Oct 14;10(10):R113. doi: 10.1186/gb-2009-10-10-r113 (PMC2784328; doi:10.1186/gb-2009-10-10-r113)
Supplement: Additional data file 3 — HIF-1 binding associations with RNA Pol II and H3K4 me3. [file gb-2009-10-10-r113-S3.PDF]

Xia_Additional3

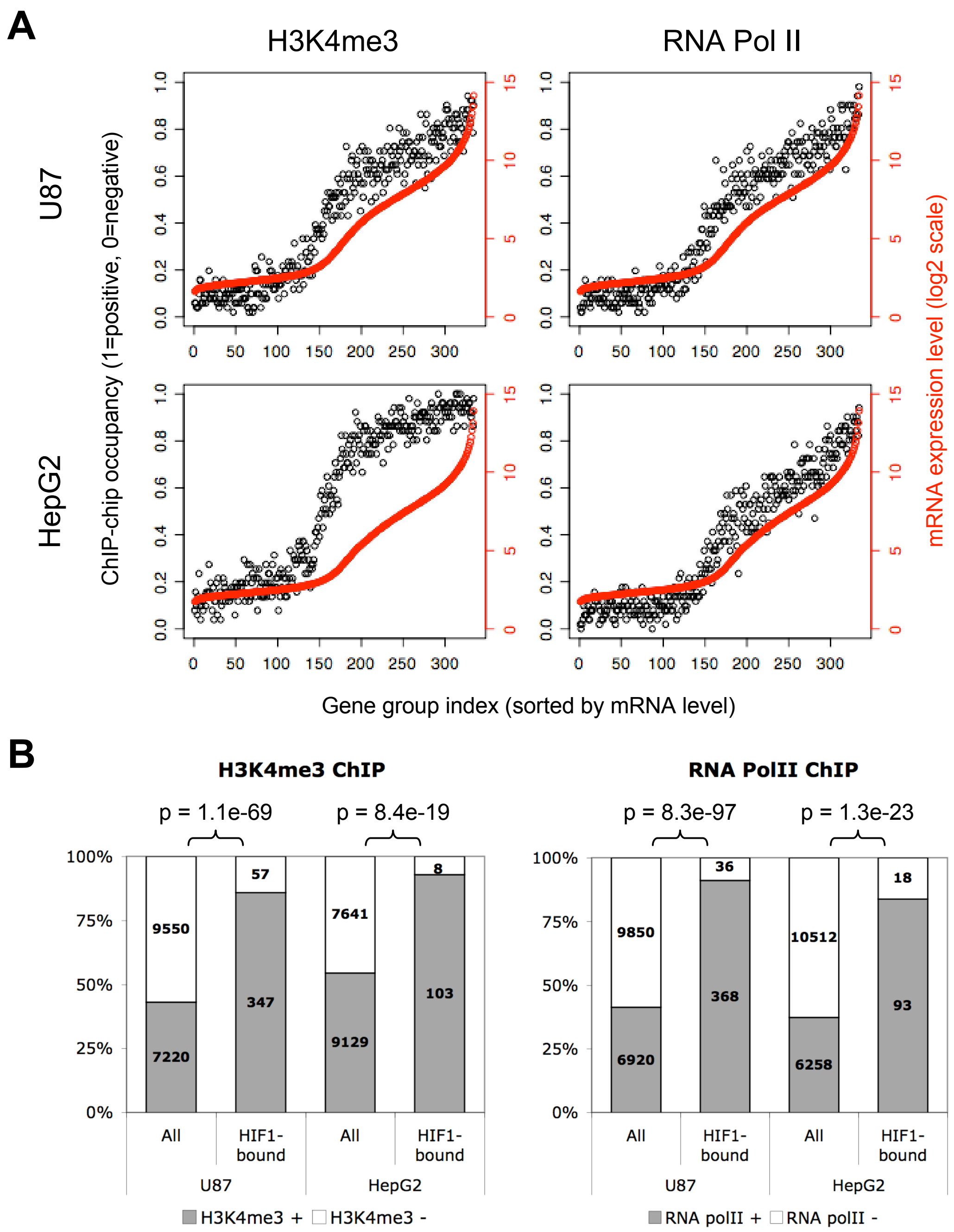

Figure S2. HIF-1 preferentially binds to loci marked with RNA Pol II and H3K4me3.

A. H3K4me3 and RNA Pol II binding percentages are correlated with mRNA expression levels. All genes were sorted by their mRNA levels and binned into sets of 100 genes. H3K4me3 or RNA Pol II occupancy percentages (1= positive, 0 = negative) of each 100 gene set (black dots in the Figure) are plotted along with their average mRNA levels (red dots).

B. HIF-1 binds to promoters that are marked with H3K4me3 or RNA Pol II under normal growth conditions. Statistical significance determined by Fisher Exact Test.
