# Supplementary material for: Preferential binding of HIF-1 to transcriptionally active loci determines cell-type specific response to hypoxia
Source: Genome Biol. 2009 Oct 14;10(10):R113. doi: 10.1186/gb-2009-10-10-r113 (PMC2784328; doi:10.1186/gb-2009-10-10-r113)
Supplement: Additional data file 4 — All common HIF-1-bound loci identified by ChIP-chip in U87 cells and HepG2 cells. [file gb-2009-10-10-r113-S4.PDF]

Supplemental Table-1. Overlapping promoter hits between U87 and HepG2 HIF-1 ChIP-chip

Supplemental Table 2. Common HIF-1 Bound loci identified by ChIP-chip in U87 cells and HepG2 cells (p-value<1e-8)

	hg18				U87 gene expr. (hr)				HepG2 gene expr. (hr)			
**Chr**	**Start**	**End**	**Gene**	**Description**	0	4	8	12	0	4	8	12
chr1	8861013	8862094	ENO1	Enolase 1	P	0.00	0.00	0.00	P	0.00	0.35	0.45
chr1	11912340	11912907	PLOD1	procollagen-lysine 1, 2-oxoglutarate 5-dioxygenase 1	P	0.00	0.00	0.29	P	0.44	0.74	1.03
chr1	11912340	11912907	KIAA2013		N/A							
chr1	33274449	33275414	AK2	adenylate kinase 2	P	0.00	0.35	0.59	P	0.00	0.00	0.00
chr1	114156240	114157293	RSBN1	round spermatid basic protein 1	P	0.59	0.56	0.44	P	1.07	1.15	1.15
chr1	201046714	201047959	JARID1B	jumonji, AT rich interactive domain 1B	P	0.60	0.81	0.89	P	1.22	1.51	1.62
chr1	234754094	234754937	LGALS8	lectin, galactoside-binding, soluble, 8 (galectin 8)	P	0.51	0.58	0.64	P	1.19	2.05	2.38
chr2	39204502	39205694	SOS1	son of sevenless homolog 1 (Drosophila)	P	1.24	1.58	1.79	P	0.00	0.74	0.82
chr2	55697615	55698826	SMEK2	SMEK homolog 2, suppressor of mek1 (Dictyostelium)	P	0.29	0.30	0.27	P	-0.68	0.00	0.00
chr2	86520960	86521968	JMJD1A	jumonji domain containing 1A	P	0.57	0.77	0.98	P	2.98	3.16	2.69
chr2	118561641	118562716	INSIG2	insulin induced gene 2	P	1.05	1.71	1.79	P	1.61	2.29	2.59
chr2	136458405	136459891	DARS	aspartyl-tRNA synthetase	P	0.00	0.00	0.00	P	0.00	0.17	0.35
chr2	176573989	176575014	KIAA1715		P	0.00	0.57	0.53	P	0.00	0.00	1.09
chr2	220149591	220150244	INHA	inhibin, alpha	A	0.00	0.00	0.00	P	0.00	0.00	1.95
chr2	220149591	220150244	OBSL1	Obscurin-like 1	P	0.00	0.00	0.00	P	0.00	0.00	0.45
chr2	238173478	238174080	RAB17	member RAS oncogene family	A	0.00	0.00	0.00	P	0.00	0.72	1.05
chr3	48569043	48570033	PFKFB4	6-phosphofructo-2-kinase/fructose-2,6-biphosphatase 4	P	1.07	1.04	1.06	A	2.81	4.05	4.27
chr3	81892351	81894167	GBE1	glucan (1,4-alpha-), branching enzyme 1	P	0.25	0.58	0.72	P	0.00	1.37	2.49
chr3	123585016	123586104	C3orf28		P	0.44	0.76	1.15	P	0.47	1.11	1.53
chr3	123585016	123586104	CCDC58	coiled-coil domain containing 58	P	0.00	0.00	0.00	A	0.00	0.00	0.00
chr3	147361508	147362505	PLOD2	procollagen-lysine, 2-oxoglutarate 5-dioxygenase 2	P	0.41	0.94	1.08	P	1.00	1.64	1.96
chr3	150425395	150426257	CP	ceruloplasmin (ferroxidase)	A	0.00	0.00	0.00	P	0.00	0.70	1.34
chr4	120440857	120441746	LOC401152	HCV F-transactivated protein 1	P	0.00	0.57	0.78	P	0.22	0.52	1.00
chr4	124537211	124538481	SPRY1	sprouty homolog 1, antagonist of FGF signaling (Drosophila)	P	2.48	1.76	1.49	P	1.51	1.57	1.79
chr4	186554143	186554761	ANKRD37	ankyrin repeat domain 37	P	2.11	2.24	2.60	A	7.24	8.04	7.78
chr5	90611548	90613506	(long range)									
chr5	130998775	130999602	RAPGEF6	Rap guanine nucleotide exchange factor (GEF) 6	P	0.00	0.00	-0.6	P	0.415	0.00	0.39
chr6	87921242	87922686	ZNF292	zinc finger protein 292	P	0.464	0.708	0.78	P	1.643	1.31	1.309
chr6	89847972	89848681	PNRC1	proline-rich nuclear receptor coactivator 1	P	0.47	1.31	1.65	P	2.22	2.48	2.54
chr6	126143221	126144298	NCOA7	nuclear receptor coactivator 7	P	0.00	0.00	0.00	P	0.81	0.91	1.26
chr7	100647583	100648319	PLOD3	procollagen-lysine, 2-oxoglutarate 5-dioxygenase 3	P	0.00	0.00	0.00	P	0.00	0.00	0.00
chr7	100647583	100648319	ZNHIT1	zinc finger, HIT type 1	P	0.00	0.00	0.00	P	0.00	-0.58	-0.55
chr7	104440016	104441154	MLL5	myeloid/lymphoid or mixed-lineage leukemia 5	P	0.00	0.00	0.00	P	1.15	0.61	0.83
chr7	127882485	127883839	HIG2	hypoxia-inducible protein 2	P	2.59	3.02	3.17	P	2.13	2.98	3.28
chr8	22510985	22512058	C8orf58		P	0.66	1.04	1.14	A	0.00	0.00	0.00
chr8	23077043	23077804	TNFRSF10D	tumor necrosis factor receptor superfamily, member 10d	P	0.00	0.00	0.23	P	0.00	0.00	1.41
chr9	6747430	6747997	JMJD2C	Jumonji domain containing 2C	A	0.00	0.00	0.00	P	0.52	0.59	0.97
chr9	35663329	35664342	CA9	carbonic anhydrase IX	P	0.00	0.56	0.82	P	1.05	3.13	4.66
chr10	70330615	70331431	DDX50	DEAD (Asp-Glu-Ala-Asp) box polypeptide 50	P	0.00	0.42	0.59	P	0.00	0.83	1.17
chr10	73703018	73704030	DDIT4	DNA-damage-inducible transcript 4	P	0.95	1.18	1.24	P	3.53	3.96	4.02
chr10	74526279	74527196	P4HA1	proline 4-hydroxylase, alpha polypeptide I	P	0.00	0.49	0.84	P	1.31	1.93	2.44
chr10	75202147	75203309	FUT11	fucosyltransferase 11	P	1.18	1.33	1.43	A	3.62	3.76	4.02
chr10	93382226	93383743	PPP1R3C	protein phosphatase 1, regulatory (inhibitor) subunit 3C	P	1.49	1.76	2.02	P	1.07	1.65	1.84
chr10	99175696	99176332	PGAM1	phosphoglycerate mutase 1 (brain)	N/A							
chr10	102095997	102096633	SCD	Stearoyl-CoA desaturase (delta-9-desaturase)	P	0.00	0.00	0.00	P	0.00	0.00	0.00
chr11	14498163	14499232	PSMA1	proteasome (prosome, macropain) subunit, alpha type, 1	P	0.00	-0.25	-0.21	P	0.00	0.00	0.00
chr11	18372355	18373091	LDHA	lactate dehydrogenase A	P	0.00	0.00	0.00	P	0.25	0.43	0.44
chr11	34083242	34084157	NAT10	N-acetyltransferase 10	P	0.00	0.00	0.00	P	0.32	0.42	0.23
chr11	73559899	73560503	PPME1	protein phosphatase methylesterase 1	P	0.00	0.72	0.93	P	0.79	0.98	1.20
chr11	73559899	73560503	C2CD3	C2 calcium-dependent domain containing 3	A	0.00	0.00	0.00	A	0.00	0.00	0.00
chr12	6512353	6514190	GAPDH	glyceraldehyde-3-phosphate dehydrogenase	P	0.00	0.00	0.00	P	0.00	0.00	0.00
chr12	10657128	10658015	MAGOHB	mago-nashi homolog B (Drosophila)	P	0.00	0.00	0.00	A	0.00	0.00	0.00
chr12	14814007	14815481	H2AFJ	H2A histone family, member J	P	0.00	0.00	0.00	P	0.00	0.00	0.00
chr12	14814007	14815481	HIST4H4	histone cluster 4, H4	N/A							
chr12	55806997	55807844	LRP1	low density lipoprotein-related protein 1	P	0.41	0.64	0.88	P	0.00	0.00	0.00
chr14	64522898	64524128	FNTB	farnesyltransferase, CAAX box, beta	P	0.00	0.00	0.00	P	0.00	0.00	0.00
chr15	73718606	73719773	IMP3	IMP3, U3 small nucleolar ribonucleoprotein, homolog (yeast)	P	0.80	0.90	1.04	P	-0.30	-0.31	-0.52
chr16	29983762	29984851	ALDOA	aldolase A	P	0.00	0.00	0.00	P	0.41	0.64	0.82
chr17	4581249	4582007	MED11	mediator complex subunit 11	P	0.00	0.00	0.00	P	0.00	0.00	0.00
chr17	7283766	7284491	FGF11	fibroblast growth factor 11	A	0.00	0.00	0.35	A	0.00	0.00	0.00
chr17	18026863	18027760	ALKBH5	alkB, alkylation repair homolog 5 (E. coli)	P	0.00	0.30	0.35	P	0.00	0.33	0.00
chr17	22644660	22645691	WSB1	WD repeat and SOCS box-containing 1	P	0.00	1.07	1.15	P	1.87	2.32	2.28
chr17	43481089	43481728	NFE2L1	nuclear factor (erythroid-derived 2)-like 1	P	0.00	0.00	0.40	P	0.58	0.99	1.57
chr17	70960766	70961264	KIAA0195		P	0.00	0.00	0.66	P	0.00	0.61	0.96
chr17	77411780	77412831	P4HB	proline 4-hydroxylase, beta polypeptide	P	0.00	0.00	0.00	P	0.00	0.00	0.00
chr17	78008892	78009920	NARF	nuclear prelamin A recognition factor	P	0.00	0.00	0.00	P	0.00	0.00	0.00
chr18	13715980	13717075	RNMT	RNA (guanine-7-) methyltransferase	P	0.79	0.81	1.03	P	1.49	2.12	2.14
chr18	13715980	13717075	C18orf19		P	0.86	0.90	0.82	P	1.19	1.69	1.56
chr18	75539914	75541060	CTDP1	TFIIF-associating CTD phosphatase 1	P	0.78	1.22	0.85	P	0.00	0.89	0.00
chr19	4919325	4920120	JMJD2B	jumonji domain containing 2B	P	0.37	0.55	0.59	P	0.60	2.11	2.32
chr19	5741493	5742370	DUS3L	Dihydrouridine synthase 3-like (S. cerevisiae)	P	0.00	0.00	0.00	A	0.00	0.77	0.65
chr19	16544182	16544877	SLC35E1	solute carrier family 35, member E1, SLC35E1	P	0.58	1.08	1.09	P	0.00	0.60	0.79
chr19	39541621	39542714	GPI	glucose phosphate isomerase	P	0.00	0.00	0.22	P	0.00	0.72	0.92
chr20	33668381	33669391	SPAG4	sperm associated antigen 4	A	0.00	0.00	0.00	P	0.00	0.00	0.00
chr22	19600925	19601597	CRKL	v-crk sarcoma virus CT10 oncogene homolog (avian)-like	P	0.47	0.40	0.00	P	1.13	1.10	1.02
chr22	22566197	22566946	MIF	macrophage migration inhibitory factor	P	0.00	0.00	0.00	P	0.00	0.00	0.00
chr22	29149013	29150577	MTP18	mitochondrial protein 18 kDa	P	0.00	0.00	0.00	P	0.70	1.02	0.69
chr22	35254617	35255631	EIF3D	eukaryotic translation initiation factor 3, subunit D	P	0.00	0.00	0.00	P	0.00	0.39	0.54
chrX	77245708	77246572	PGK1	phosphoglycerate kinase 1	P	0.00	0.00	0.00	P	0.48	1.36	1.74

P: preser A: absent
N/A: no probe on the microarray

>1 1 0.5 0 -0.5 -1

not significant (P>0.01)
